# Hectd3 promotes pathogenic Th17 lineage through Stat3 activation and Malt1 signaling in neuroinflammation

**DOI:** 10.1038/s41467-019-08605-3

**Published:** 2019-02-11

**Authors:** Jonathan J. Cho, Zhiwei Xu, Upasana Parthasarathy, Theodore T. Drashansky, Eric Y. Helm, Ashley N. Zuniga, Kyle J. Lorentsen, Samira Mansouri, Joshua Y. Cho, Mariola J. Edelmann, Duc M. Duong, Torben Gehring, Thomas Seeholzer, Daniel Krappmann, Mohammad N. Uddin, Danielle Califano, Rejean L. Wang, Lei Jin, Hongmin Li, Dongwen Lv, Daohong Zhou, Liang Zhou, Dorina Avram

**Affiliations:** 1 Department of Anatomy and Cell Biology, College of Medicine, University of Florida, Gainesville, FL 32610 USA; 2 Department of Medicine, College of Medicine, University of Florida, Gainesville, FL 32610 USA; 3 Department of Microbiology and Cell Science, University of Florida, Gainesville, 32611 Florida USA; 4 Center for Neurodegenerative Diseases, Emory University School of Medicine, Atlanta, 30322 GA USA; 5 Department of Biochemistry, Emory University School of Medicine, Atlanta, 30322 GA USA; 6 Research Unit Cellular Signal Integration, Institute of Molecular Toxicology and Pharmacology, Helmholtz Zentrum München - German Research Center for Environmental Health, Ingolstaedter Landstrasse 1, 85764 Neuherberg, Germany; 7 Department of Immunology and Microbial Disease, Albany Medical Center, Albany, 12208 NY USA; 8 UF Health Cancer Center, University of Florida, Gainesville, FL 32610 USA; 9 Wadsworth Center, New York State Department of Health, 120 New Scotland Ave, Albany, NY 12208 USA; 10 Department of Pharmacodynamics, College of Pharmacy, University of Florida, Gainesville, FL 32610 USA; 11 Department of Infectious Diseases and Immunology, College of Veterinary Medicine, University of Florida, Gainesville, FL 32608 USA

## Abstract

Polyubiquitination promotes proteasomal degradation, or signaling and localization, of targeted proteins. Here we show that the E3 ubiquitin ligase Hectd3 is necessary for pathogenic Th17 cell generation in experimental autoimmune encephalomyelitis (EAE), a mouse model for human multiple sclerosis. Hectd3-deficient mice have lower EAE severity, reduced Th17 program and inefficient Th17 cell differentiation. However, Stat3, but not RORγt, has decreased polyubiquitination, as well as diminished tyrosine-705 activating phosphorylation. Additionally, non-degradative polyubiquitination of Malt1, critical for NF-κB activation and Th17 cell function, is reduced. Mechanistically, Hectd3 promotes K27-linked and K29-linked polyubiquitin chains on Malt1, and K27-linked polyubiquitin chains on Stat3. Moreover, Stat3 K180 and Malt1 K648 are targeted by Hectd3 for non-degradative polyubiquitination to mediate robust generation of RORγt^+^IL-17A^hi^ effector CD4^+^ T cells. Thus, our studies delineate a mechanism connecting signaling related polyubiquitination of Malt1 and Stat3, leading to NF-kB activation and RORγt expression, to pathogenic Th17 cell function in EAE.

## Abstract (web summary)

Ubiquitination may control protein stability or function. Here the authors show that an ubiquitination enzyme, Hectd3, ubiquitinates Stat3 and Malt1 to modulate their function but not degradation in T cells, and thereby promoting the differentiation of pathogenic Th17 cells and susceptibility to a mouse model of multiple sclerosis.

## Introduction

T helper 17 (Th17) cells are a distinct subset of CD4^+^ T cells that mediate host defense against specific pathogens and have essential functions in many autoimmune diseases^1^. Th17 cells have recently come into sharp focus in relation with their role in autoimmunity, including experimental autoimmune encephalomyelitis (EAE)^2,3^, multiple sclerosis (MS)^4,5^, collagen-induced arthritis^6^, Crohn’s disease^7^, and rheumatoid arthritis^8^. Key cytokines and transcription factors are critical for the differentiation and function of Th17 cells. Following T cell receptor (TCR) stimulation, the transcription factors BATF^9^ and IRF4^10^ are upregulated and cooperatively pre-pattern the chromatin landscape for Th17 cell specification^11^. In addition, the cytokines IL-6 and TGF-β are required for initiation of Th17 differentiation^12^. Specifically, IL-6 signaling engenders phosphorylation and activation of Stat3, which is another key transcription factor in Th17 cell differentiation^13–15^. The master transcription factor controlling Th17 cell identity, RORγt, acts synergistically with activated Stat3 to maximize the transcription of *Il17a*, *Il17f*, *Il23r*, and *Ccr6*^11,14,16^. The terminal differentiation and induction of pathogenic Th17 cells requires IL-23R^17^. IL-23R signals through Stat3, to maintain and stabilize the expression of RORγt, IL-17A, and IL-23R itself^17^. In addition, in MS and its mouse model EAE^2,18,19^, IL-23R signaling drives the co-expression of the highly pro-inflammatory cytokines GM-CSF and IFNγ through RORγt, activated Stat3, and Blimp-1^17–20^.

Ubiquitination is known to play a key role in the regulation of Th17 cells. RORγt was shown to be targeted for proteasomal degradation via K48-linked polyubiquitination by the HECT E3 ubiquitin ligase Itch to suppress colonic inflammation^21^. RORγt is additionally regulated by ubiquitination via TRAF5 through K63-linked polyubiquitin chains, which promotes its stability and IL-17A production in in vitro polarized human CD4^+^ T cells^22^. Furthermore, the UBR box E3 ligase UBR5 and the deubiquitinase DUBA regulate RORγt-associated colonic inflammation in a cell-intrinsic manner^23^. In addition to RORγt, Stat3 was found to be ubiquitinated and degraded in Th17 cells by the E3 ubiquitin ligase SLIM/PDLIM2 also in a cell-intrinsic manner^24^. TRIM21 also influences T cell differentiation by targeting IRF3 to inhibit ex vivo Th1 and Th17 differentiation in CD4^+^ T cells isolated from inflammatory bowel disease (IBD) patients^25^. Numerous deubiquitinases have also been shown to influence Th17 cells. USP4^26^, USP15^27^, and USP17^28^ promote RORγt activity in in vitro polarized Th17 cells and enhance the recruitment of the co-activator SRC1 or stabilize RORγt via deubiquitination. USP18 promotes Th17 cell differentiation by limiting production of IL-2 and Stat5 activation through deubiquitination of TAK1 in EAE^29^. However, the E3 ubiquitin ligase mediating TAK1 ubiquitination is unknown. Thus, no E3 ubiquitin ligase has been shown to regulate Th17 cells in a cell-intrinsic manner in vivo through a signaling type of polyubiquitination that does not target its substrate for proteasomal degradation. Given the regulatory roles of E3 ubiquitin ligases, this is of outstanding importance in the context of autoimmunity in EAE and MS.

The HECT E3 ubiquitin ligase Hectd3 was shown to promote non-K48-linked polyubiquitination on Malt1, enhancing its stability in cancer cell lines^30^. In addition, Hectd3 was found to ubiquitinate several caspases, regulating survival of cancer cell lines^31,32^. Recently, Hectd3 was found to promote type I interferon (IFN) response in bone-marrow-derived macrophages through polyubiquitination of TRAF3, and this correlated with altered antibacterial response^33^. Malt1, an essential component of the Carma1-Bcl10-Malt1 (CBM) complex^34^, was found to be essential for Th17 pathogenicity in EAE through promoting nuclear translocation of p65 and inhibiting nuclear translocation of RelB^35,36^. Given that Hectd3 was detected in the molecular core regulating Th17 cell differentiation^11^, we asked the questions whether Hectd3 ubiquitinates Malt1 to regulate Th17 cell differentiation during EAE and whether there are additional Malt1-independent targets by which Hectd3 controls pathogenic Th17 cell differentiation.

Here we show that Hectd3 promotes pathogenic Th17 cell differentiation in EAE, in a cell-intrinsic manner, through non-degradative polyubiquitination of Malt1 and Stat3. We find that EAE severity is attenuated in *Hectd3*^−/−^ mice, which correlates with diminished IL-17A and GM-CSF production, RORγt levels, and reduced phosphorylated (p)Stat3 Y705 in *Hectd3*^−/−^ CD4^+^ T cells. Related to this phenotype, Stat3 polyubiquitination is decreased in *Hectd3*^−/−^ CD4^+^ T cells, while that of RORγt remains unchanged. In addition, we show that Hectd3 promotes non-degradative K27 polyubiquitination at Stat3 K180, which is essential for robust generation of RORγt^+^IL-17A^hi^ Th17 cells. In addition, Malt1 is targeted for non-degradative K27 and K29 polyubiquitination by Hectd3 at K648, which is also essential for RORγt^+^IL-17A^hi^ Th17 cell generation. Our study thus demonstrates that non-degradative polyubiquitination of Malt1 and Stat3 by Hectd3, leading to NF-kB activation and RORγt upregulation, respectively, delineates a mechanism connecting ubiquitination to Th17 differentiation and function in the context of autoimmunity and EAE.

## Results

### Hectd3 KO CD4^+^ T cells have altered Th17 polarization

**Fig. 1:**
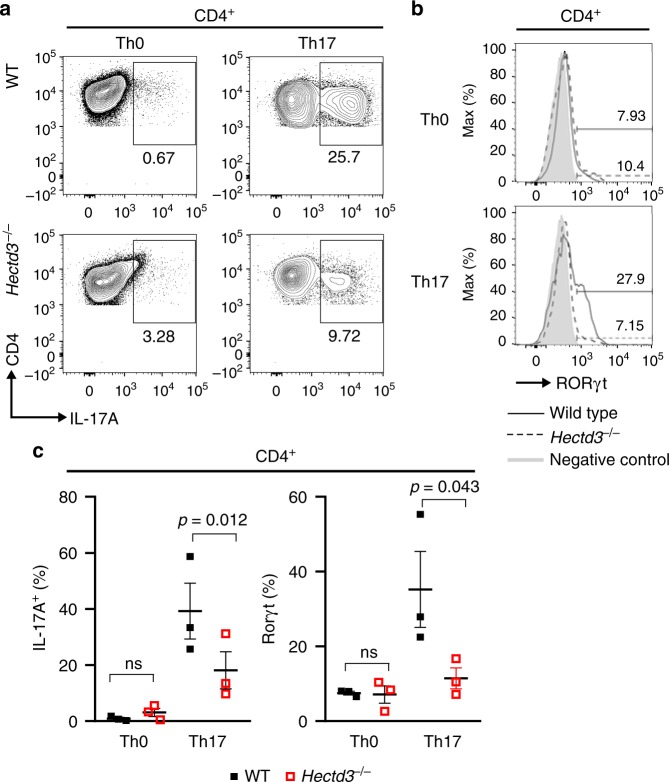
Defective Th17 polarization of Hectd3-deficient CD4^+^ T cells ex vivo. **a** Representative flow cytometry analysis of intracellular IL-17A from in vitro polarized *Hectd3*^−/−^ and wild-type (WT) CD4^+^ T cells under Th0 or Th17 conditions, *n* = 3 per group, from three independent experiments. **b** Representative flow cytometry analysis of RORγt in in vitro polarized *Hectd3*^−/−^ and WT CD4^+^ T cells under Th0 or Th17 conditions, *n* = 3 per group, from three independent experiments. Th0 or Th17 conditions are described in Material and methods. **c** Quantification of **a** and **b**. Data are presented as mean ± SEM; *p* values were determined using Student’s *t* test. Source data are provided as a Source Data file. Gating strategy is shown in Supplementary Fig. 9

Malt1 was identified as a target for Hectd3-mediated non-degradative polyubiquitination in cancer cell lines^30^ and plays a role in Th17 cell-associated pathogenicity in EAE^35^. Additionally, Hectd3 was recently detected in the gene network regulating Th17 lineage, as reported by Ciofani et al.^11^ (Supplementary Fig. 1a, data from Ciofani et al.^11^). We thus investigated Hectd3 expression in T helper subsets and found that Hectd3 protein was expressed in naïve and in vitro polarized CD4^+^ T cells, including in the Th17 subset (Supplementary Fig. 1b). Additionally, Hectd3 was expressed in the main immune populations, including T, B, and myeloid cells (Supplementary Fig. 1c). *Hectd3*^*−*^^/−^ CD4^+^ T cells polarized similarly to wild-type (WT) CD4^+^ T cells in vitro under Th1, Th2, and Treg conditions and showed no differences in the Th1 transcription factor T-bet and cytokine IFNγ, Th2 transcription factor Gata3 and cytokine IL-4, and Treg transcription factor Foxp3, respectively (Supplementary Fig. 1d-f). However, under Th17 polarizing conditions, there was a significant reduction in the generation of RORγt^+^ and IL-17A^+^ T helper cells in the absence of Hectd3 (Fig. 1a–c). CD4^+^, CD8^+^, and Foxp3^+^ regulatory T cells had similar frequencies and numbers in peripheral lymphoid organs of *Hectd3*^−/−^ and WT mice at steady state (Supplementary Fig. 1g-h), and thymic populations were also equivalent (Supplementary Fig. 1i). These results show that while in the absence of Hectd3, CD4^+^ and CD8^+^ T cell subsets are unaltered at steady state and CD4^+^ T cells polarize normally to Th1, Th2, and Treg cells ex vivo*, Hectd3*^−/−^ CD4^+^ T cells polarized poorly under Th17 conditions.

### Hectd3 KO mice have attenuated EAE severity

**Fig. 2:**
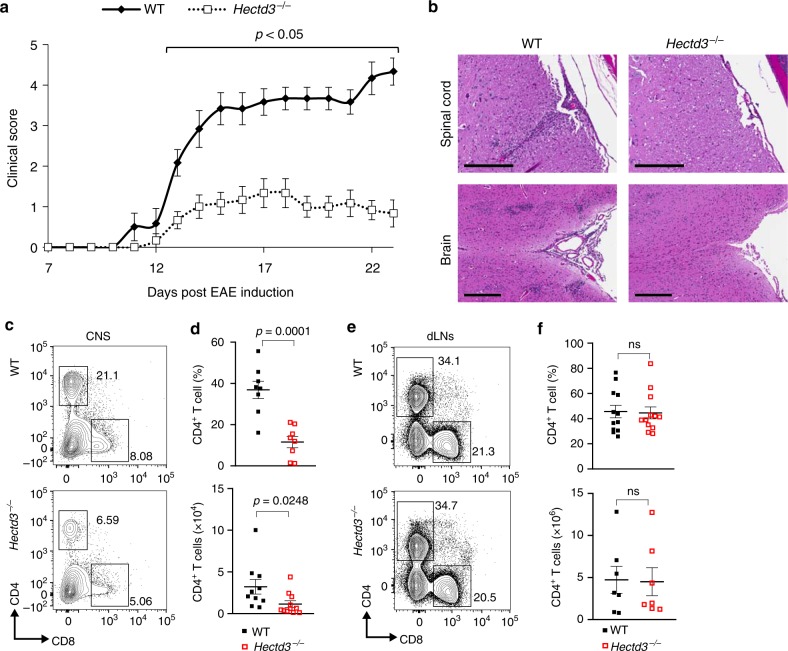
Hectd3-deficient mice have attenuated EAE severity with reduced CD4^+^ T cell infiltration in the CNS. **a** EAE disease scores (mean ± SEM) of *Hectd3*^−/−^ (open) and wild-type (WT) (filled) mice following EAE induction, *n* = 6 per group, from three independent experiments. EAE induction was conducted as described in Material and methods. Description of clinical scores for EAE is presented in Material and methods. **b** Representative spinal cord and brain hematoxylin and eosin staining of *Hectd3*^−/−^ and WT mice 16 days following EAE induction; black bars represents 200 μm for spinal cord sections and 300 μm for brain sections; *n* = 4 per group, from three independent experiments. **c** Flow cytometry analysis of CD4^+^ and CD8^+^ T cells in the CNS of *Hectd3*^−/−^ and WT mice 13 days following EAE induction, *n* = 8 per group from three independent experiments. **d** Frequencies and absolute numbers (mean ± SEM) of the CNS CD4^+^ T cells in *Hectd3*^-−/−^ and WT mice, 13 days following EAE induction. **e** Flow cytometry analysis of CD4^+^ and CD8^+^ T cells in draining lymph nodes (dLNs) of *Hectd3*^−/−^ and WT mice 13 days following EAE induction; *n* = 10 per group from three independent experiments. **f** Frequencies and absolute numbers (mean ± SEM) of CD4^+^ T cells in the dLNs of *Hectd3*^−/−^ and WT mice, 13 days following EAE induction, *n* = 7 per group from three independent experiments. **c**–**f**, Data (*n* = 6–10) are representative of three independent experiments and are presented as mean ± SEM; *p* value was obtained using Mann–Whitney two-tailed test for the EAE clinical scores and Student’s two-tailed *t* test for all other data. Source data are provided as a Source Data file. Gating strategy is shown in Supplementary Fig. 9

Given the altered ex vivo Th17 polarization in the absence of Hectd3, we investigated the role of Hectd3 in EAE pathogenesis, which is predominantly driven by a pathogenic Th17 response. Upon EAE induction, *Hectd3*^−/−^ mice (Supplementary Fig. 1j) developed less severe EAE, with diminished clinical scores compared to WT mice (Fig. 2a). Histopathological H&E examination of the CNS showed reduced immune cell infiltration in *Hectd3*^−/−^ mice at the peak of disease, compared to WT mice (Fig. 2b). Thus, absence of Hectd3 causes reduced EAE severity, associated with reduced infiltration of immune cells in the CNS.

### The Th17 program is defective in Hectd3 KO mice during EAE

**Fig. 3:**
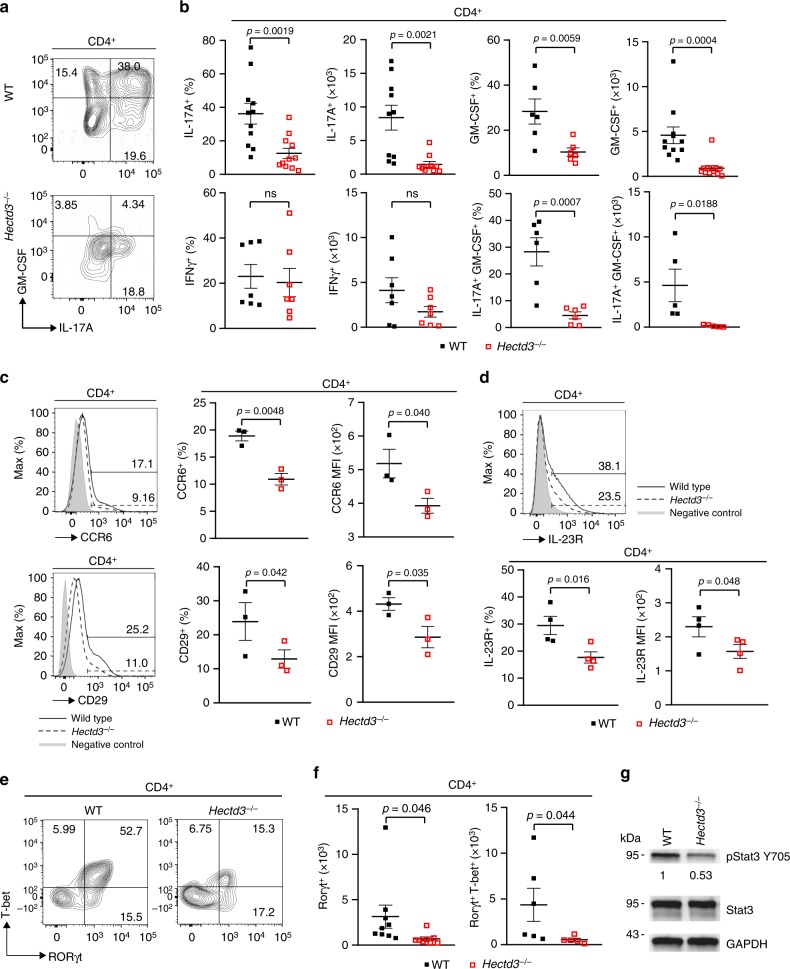
Th17 cell program and pStat3 Y705 are defective in Hectd3-deficient T helper cells during EAE. **a** Representative flow cytometry analysis of intracellular IL-17A and GM-CSF in CD4^+^ T cells from the CNS of *Hectd3*^−/−^ and wild-type (WT) mice, 13 days following EAE induction. **b** Frequencies and absolute numbers of IL-17A^+^, GM-CSF^+^, IFNγ^+^, and IL-17A^+^GM-CSF^+^ CD4^+^ T cells in the CNS of *Hectd3*^−/−^ and WT mice, 13 days following EAE induction; *n* = 5 per group, from three independent experiments. **c**, **d** Representative flow cytometry histograms, frequencies, and MFIs of CCR6, CD29, and IL-23R in draining lymph node CD4^+^ T cells from the indicated groups of mice 10 days following EAE induction; data (*n* = 6) are representative of three independent experiments. **e** Representative flow cytometry analysis of RORγt^+^ T-bet^+^ CD4^+^ T cells in the CNS of *Hectd3*^−/−^ and WT mice, 13 days following EAE induction, *n* = 6 per group from three independent experiments. **f** Absolute numbers of RORγt^+^ CD4^+^ T cells and RORγt^+^ T-bet^+^ CD4^+^ T cells in the CNS of *Hectd3*^−/−^ and WT mice, 13 days following EAE induction. **g** Representative immunoblot of pStat3 Y705 and Stat3 protein level in CD4^+^ T cells isolated from draining lymph nodes of *Hectd3*^−/−^ and WT mice, 13 days following EAE induction, from five independent experiments. Data are mean ± SEM; *p* value was obtained from Student’s *t* test. Source data are provided as a Source Data file. Gating strategy is shown in Supplementary Fig. 9

Given the reduced infiltration of immune cells in the CNS and reduced IL-17A in the absence of Hectd3 during Th17 polarization, we further examined the CD4^+^ T cells and the associated cytokines in the CNS and draining lymph nodes (dLNs) of EAE *Hectd3*^−/−^ and WT control mice. The frequencies and absolute numbers of CD4^+^ T cells, as well as that of IL-17A^+^GM-CSF^+^CD4^+^ T cells, were reduced in the CNS of *Hectd3*^−/−^ mice (Fig. 2c, d and Fig. 3a, b). While the frequencies and absolute numbers of CD4^+^ T cells from EAE *Hectd3*^−/−^ mice were not reduced in the dLNs (Fig. 2e, f), frequencies and absolute numbers of IL-17A^+^ and GM-CSF^+^ CD4^+^ T cells were lower (Supplementary Fig. 2a-b), and no change was observed in IFNγ^+^ cells (Supplementary Fig. 2a, b). In addition, the levels of key CNS homing markers, CCR6^37^ and CD29^38^, were reduced on CD4^+^ T cells of EAE *Hectd3*^−/−^ mice compared to WT controls (Fig. 3c). Moreover, the level of IL-23R, essential for terminal differentiation of pathogenic Th17 cells^17^, was also reduced on CD4^+^ T cells from EAE *Hectd3*^−/−^ mice (Fig. 3d). Given the overall reduction in Th17 program, including cytokines and the pathogenicity marker IL-23R, all known to be transcriptionally regulated by RORγt^11,13,17,39^, we evaluated the level of the Th17 transcription factor RORγt in CNS-infiltrating and dLN CD4^+^ T cells from *Hectd3*^−/−^ mice during EAE. We found that the frequencies and absolute numbers of CNS-infiltrating and dLN RORγt^+^ CD4^+^ T cells in EAE *Hectd3*^−/−^ mice were reduced compared to WT mice (Fig. 3e, f, Supplementary Fig. 2c). We found that the frequencies and absolute numbers of CNS-infiltrating RORγt^+^T-bet^+^ CD4^+^ T cells, a postulated highly pathogenic CD4^+^ T cell population^1^, were reduced in EAE *Hectd3*^−/−^ mice compared to WT (Fig. 3e, f, Supplementary Fig. 2c). Since Hectd3 protein is also expressed in myeloid cells, we examined the frequency of myeloid leukocytes in the CNS of WT and *Hectd3* KO EAE mice and found no difference (Supplementary Fig. 2d-e). Overall, these results show that Hectd3 controls the Th17 cell pathogenic program in EAE.

### pStat3 Y705 is diminished in Hectd3 KO CD4^+^ T cells in EAE

Since the level of RORγt was reduced in EAE *Hectd3*^−/−^ CNS-infiltrating and dLN CD4^+^ T cells and Stat3 is known to be essential for RORγt expression and Th17 pathogenicity^15^, we examined the pStat3 Y705 and total Stat3 in CD4^+^ T cells from EAE *Hectd3*^−/−^ mice. pStat3 Y705 was reduced in the dLN CD4^+^ T cells from EAE *Hectd3*^−/−^ mice (Fig. 3g); however, total Stat3 remained unaltered (Fig. 3g), thus implicating Hectd3 in the control of Stat3 phosphorylation and activation.

### Hectd3-deficient Th17 cells have reduced pathogenicity

**Fig. 4:**
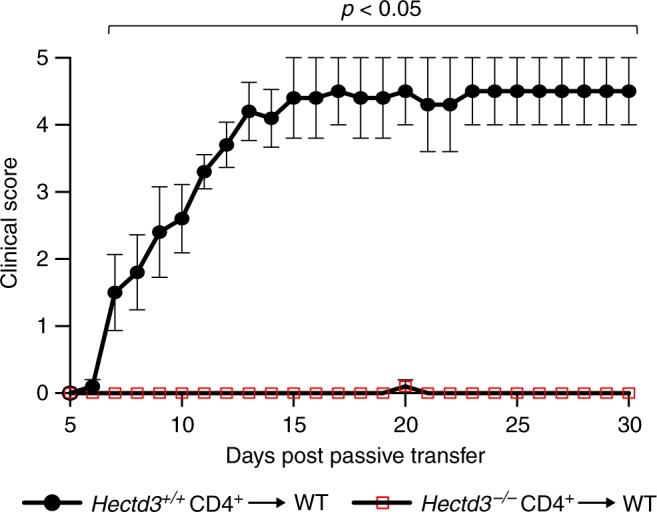
Hectd3-deficient Th17 cells have reduced pathogenicity. EAE disease scores (mean ± SEM) of WT recipients transferred with in vitro IL-23 re-stimulated CD4^+^ T cells from either *Hectd3*^−/−^ or WT mice, induced with EAE, as indicated in Materials and methods. Details on induction of passive EAE are provided in Materials and methods. Clinical scoring of recipient mice was performed using criteria listed in Material and methods, 5 days following adoptive/passive transfer of in vitro reactivated CD4^+^ T cells into recipient mice; *n* = 5 per group from two independent experiments. Mann–Whitney two-tailed test was used for statistical analysis of EAE clinical scores. Source data are provided as a Source Data file

We found that RORγt and IL-17A were reduced in *Hectd3*^−/−^ CD4^+^ T cells under Th17-polarizing conditions ex vivo (Fig. 1a, b), suggesting that Hectd3 plays a cell-intrinsic role in upregulation of RORγt, and production of IL-17A in these cells. We thus evaluated the *Hectd3*^−/−^ Th17 pathogenicity in vivo, by adoptively transferring in vitro Th17 conditioned, re-stimulated *Hectd3*^−/−^ or WT CD4^+^ T cells into WTmice. We found that *Hectd3*^−/−^ CD4^+^ T cell pathogenicity in this EAE passive transfer model was completely abolished (Fig. 4). These data demonstrate that Hectd3 deficiency causes a cell-intrinsic defect in Th17 cell pathogenicity that is responsible for the attenuation of EAE in *Hectd3*^−/−^ mice.

### Activation of Hectd3 KO CD4^+^ T cells is unaltered in EAE

We next sought to determine if activation and proliferation of *Hectd3*^−/−^ CD4^+^ T cells was affected during EAE. The results show that the activation markers CD44, CD62L, CD69, and CD25 were similar between *Hectd3*^−/−^ and WT dLN CD4^+^ T cells from EAE mice (Supplementary Fig. 2f-h). The frequency of cells entering the cell cycle, determined by the nuclear antigen Ki-67, was also similar (Supplementary Fig. 2i). These data suggest that T cell priming of *Hectd3*^−/−^ CD4^+^ T cells was unaffected during EAE.

### Malt1 is polyubiquitinated by Hectd3 in CD4^+^ T cells in EAE

**Fig. 5:**
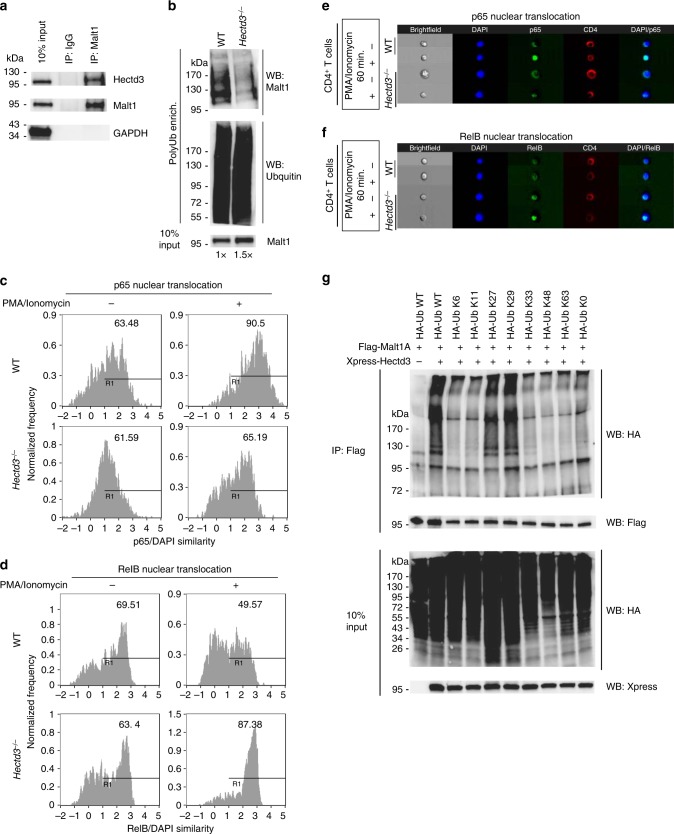
Hectd3 interacts and promotes non-degradative K27- and K29-linked polyubiquitination on Malt1 and NF-κB activation in CD4^+^ T cells. **a** Immunoblot of Hectd3, Malt1, and GAPDH following Malt1 or IgG immunoprecipitation of protein extracts from draining lymph node CD4^+^ T cells of WT mice 13 days following EAE induction. **b** Immunoblot of Malt1 following polyubiquitinated protein enrichment of extract from draining lymph node CD4^+^ T cells of wild-type *Hectd3*^−/−^ or (WT) mice 13 days following EAE induction. About 1.5× the amount of total protein from *Hectd3*^−/−^ CD4^+^ T cells, compared to WT CD4^+^ T cells, was used to normalize for the reduction in Malt1 protein level in *Hectd3*^−/−^ CD4^+^ T cells. At least 700 μg of total protein from mouse primary CD4^+^ T cells were enriched for ubiquitinated protein with Ubiquitinated Protein Enrichment Kit or Anti-Ub TUBE2, Agarose following manufacturer’s protocol. **c**, **d** Percentage of p65 or RelB nuclear translocation using p65/DAPI similarity analysis from ImageStream, in CD4^+^ T cells isolated from draining lymph nodes of *Hectd3*^−/−^ and WT mice, 13 days following EAE induction. **e**, **f** ImageStream fluorescence imaging of PMA/ionomycin-induced p65 nuclear translocation (**e**) or RelB (**f**) in CD4^+^ T cells isolated from draining lymph nodes of *Hectd3*^−/−^ or WT mice, 13 days following EAE induction. **g** Immunoblot of HA, Flag, and Xpress following two-step Flag immunoprecipitation of protein extracts from HEK293T cells co-transfected with the indicated HA-Ub K only mutants, Flag-Malt1A, and Xpress-Hectd3. HA-Ub K only mutant denote that the only lysine in the HA-tagged ubiquitin is at the indicated residue, and all other lysine residues are mutated to arginine. HA-Ub K0 mutant indicate that all seven lysine residues are mutated to arginine. **a**–**g** Immunoblots, ImageStream similarity analyses, and ImageStream fluorescence imaging are representative of at least three independent experiments. Source data are provided as a Source Data file

Since Hectd3 was previously shown to interact with and polyubiquitinate Malt1 in cancer cell lines^30^, we examined Hectd3 and Malt1 interaction in CD4^+^ T cells from EAE mice, and found that Hectd3 associates with Malt1 in CD4^+^ T cells during EAE (Fig. 5a). We next examined Malt1 polyubiquitination in *Hectd3*^−/−^ CD4^+^ T cells from EAE mice. We used 1.5 times more total protein from *Hectd3*^−/−^ CD4^+^ T cells versus WT, to normalize for the slight reduction in Malt1 protein level in *Hectd3*^−/−^ CD4^+^ T cells, and found that Malt1 polyubiquitination was reduced in *Hectd3*^−/−^ CD4^+^ T cells during EAE (Fig. 5b). Thus, Hectd3 interacts and promotes polyubiquitination of Malt1 in T helper cells from EAE mice.

### Nuclear p65 and RelB in Hectd3 KO CD4^+^ T cells in EAE

NF-κB p65 nuclear translocation was reduced, while RelB nuclear translocation was increased in *Malt1*^−/−^ CD4^+^ T cells from EAE mice^35^. We thus tested the nuclear translocation of p65 and RelB in *Hectd3*^−/−^ CD4^+^ T cells from EAE mice. p65 nuclear translocation was decreased, while RelB nuclear translocation was increased in CD4^+^ T cells isolated from EAE *Hectd3*^−/−^ mice (Fig. 5c–f), similar to *Malt1*^−/−^ CD4^+^ T cells^35^. Thus, diminished Hectd3-mediated polyubiquitination of Malt1 correlates with decreased p65 nuclear translocation but increased RelB nuclear translocation.

### Hectd3 promotes K27/K29-linked polyubiquitin chains on Malt1

Hectd3 was shown to mediate non-K48-linked polyubiquitination on Malt1 in cancer cell lines; however, the precise type of ubiquitin chain is not known^30^. Using transfection of lysine (K) only ubiquitin mutants where all seven lysine residues were mutated to arginine, except the indicated lysine residue, and two-step immunoprecipitation of Flag-tagged Malt1A, a splice variant of Malt1 expressed in activated CD4^+^ T cells^40^, we found that the HA-Ub K27 only and HA-Ub K29 only mutants were sufficient for Hectd3-mediated ubiquitination of Malt1A (Fig. 5g). Conversely, Hectd3-mediated polyubiquitination was abolished in HA-Ub K27R and HA-Ub-K29R mutants but not in the WT HA-Ub, HA-Ub K11R, and HA-Ub K48R, while HA-Ub K63R mutant showed variability (Supplementary Fig. 3). Thus, based on the results with ubiquitin K only and K to R mutants, we concluded that Hectd3 predominantly promotes K27- and K29-linked polyubiquitin chains on Malt1.

### Malt1 K648 ubiquitination by Hectd3 in Th17 cell generation

**Fig. 6:**
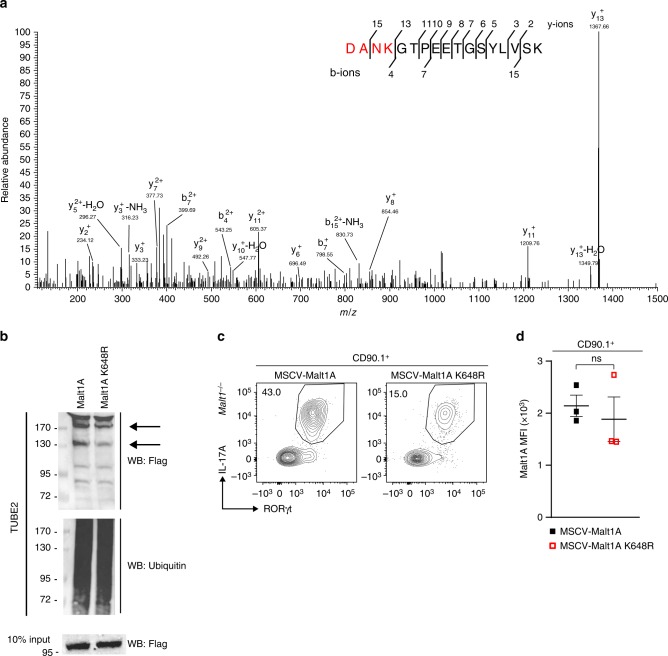
Ubiquitination of Malt1A K648 by Hectd3 is essential for RORγt^+^IL-17A^hi^ Th17 cell generation. **a** HEK293T cells were co-transfected with HA-Ub, Flag-Malt1A, and Xpress-Hectd3. Extracts were immunoprecipitated with anti-Flag antibodies, followed by trypsin digestion and tandem mass spectrometry, as described in Material and methods. A fragmentation spectrum of ubiquitinated DANKGTPEETGSYLVSK peptide (ubiquitinated K648 residue) of Malt1A. Parent ion corresponding to DANkGTPEETGSYLVSK peptide mass has been subjected to higher-energy collisional dissociation in mass spectrometer. The detected b- and y-fragment ion series have been annotated. **b** Representative immunoblot of Flag and ubiquitin following polyubiquitinated protein enrichment of extracts using TUBE2 from CD90.1+ sorted EL4 cells transduced with MSCV-CD90.1-Flag-Malt1A or MSCV-CD90.1-Flag-Malt1A K648R retrovirus. **c** Flow cytometry analysis of intracellular IL-17A and intranuclear RORγt in CD90.1^+^ *Malt1*^−/−^ CD4^+^ T cells transduced with indicated retrovirus and in vitro polarized under Th17 conditions. Representative of three independent experiments. Gating strategy was first on CD90.1^+^ T cells. **d** MFI of Malt1A in CD90.1^+^ *Malt1*^−/−^CD4^+^ T cells transduced with indicated retroviruses and in vitro polarized under Th17 condition. Data (*n* = 6) are mean of three independent experiments and are presented as mean ± SEM; *p* value was obtained from Student’s *t* test. Source data are provided as a Source Data file

We further conducted tandem mass spectrometry analysis of Flag-immunoprecipitated Malt1A complex from HEK293T cells co-transfected with HA-Ub, Flag-Malt1A, and with or without Xpress-Hectd3, and identified Malt1A K648 as having the K-ε-GG residue only in conditions with Hectd3 transfection (Fig. 6a). To demonstrate that Malt1A K648 is indeed the target for Hectd3-mediated polyubiquitination, we generated by site-directed mutagenesis Flag-Malt1A K648R mutant retroviruses, and transduced EL4 T cells. We found that Malt1A K648R mutant had reduced polyubiquitination (Fig. 6b). In addition, using an ex vivo reconstitution assay, we found that MSCV-MALT1A K648R mutant generated a lower frequency of RORγt^+^IL-17A^hi^ population compared to WT MSCV-MALT1A in transduced *Malt1*^−/−^ CD4^+^ T cells polarized in Th17 conditions (Fig. 6c). The expression level of Malt1A and Malt1A K648R in transduced *Malt1*^−/−^ CD4^+^ cells polarized in Th17 conditions was equal (Fig. 6d). These data show that Hectd3-mediated ubiquitination of Malt1A K648 is essential for generation of Th17 cells with robust RORγt and IL-17A production.

### K648 in Malt1A paracaspase activity and CBM in Jurkat cells

Since ubiquitination of Malt1 has been shown to dictate Malt1 paracaspase activity and CBM complex formation^41,42^, we sought to characterize the role of K648 in relation to these signaling properties of Malt1. CYLD^43^ and HOIL-1^44–46^ are two of the well-characterized substrates of Malt1 in lymphocyte signaling. To determine the effect of Malt1A ubiquitination at K648 on Malt1A substrate cleavage activity, we transduced MALT1KO Jurkat cells with MSCV-Malt1A WT or MSCV-Malt1A K648R and then stimulated the reconstituted cells with αCD3/αCD28. We observed no difference in the cleavage of CYLD and HOIL-1 between MALT1KO Jurkat cells transduced with Malt1A WT or Malt1A K648R (Supplementary Fig. 4a). We next examined CBM complex formation in MALT1KO Jurkat cells transduced with Malt1A WT or Malt1A K648R and found no difference in CARMA1 and BCL10 association in the presence of Malt1A WT or Malt1A K648R (Supplementary Fig. 4b). Thus, Malt1A K648 does not affect Malt1 substrate cleavage and CBM complex formation in Jurkat cells, suggesting that either Malt1A K648 may control generation of RORγt^+^IL17^hi^ Th17 cells through an undiscovered mechanism, or the signaling components and mechanisms of regulation are different in Th17 cells compared to Jurkat cells.

### Hectd3 polyubiquitinates Stat3 in CD4^+^ T cells in EAE

**Fig. 7:**
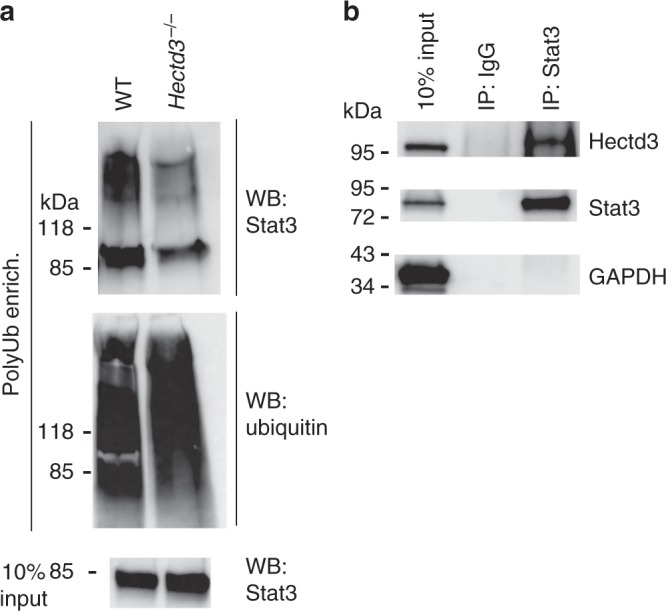
Hectd3 interacts with and promotes Stat3 polyubiquitination. **a** Immunoblot of Stat3 and ubiquitin following polyubiquitinated protein enrichment of extracts from dLN CD4^+^ T cells of *Hectd3*^*−/−*^ or wild-type (WT) mice, 13 days following EAE induction. **b** Immunoblot of Hectd3 and Stat3 following Stat3 or IgG immunoprecipitation of protein extracts from dLN CD4^+^ T cells of WT mice 13 days following EAE induction. **a**, **b** Immunoblots are representative of three independent experiments. Source data are provided as a Source Data file

Given the reduction in pStat3 Y705 in CD4^+^ T cells of EAE *Hectd3*^*−*^^/−^ mice, we tested whether Hectd3 polyubiquitinates Stat3. Indeed, Stat3 polyubiquitination was reduced in *Hectd3*^−/−^ CD4^+^ T cells during EAE without overall change in its level (Fig. 7a and Fig. 3g). Additionally, Hectd3 and Stat3 were present in the same complex in CD4^+^ T cells from EAE mice (Fig. 7b), as well as in naïve CD4^+^ T cells, while treatment with IL-6 did not influence their association (Supplementary Fig. 5). We further examined ubiquitination of RORγt in EAE *Hectd3*^*−/−*^ and WT CD4^+^ T cells and did not observe any difference in polyubiquitination (Supplementary Fig. 6). Thus, these results demonstrate that Hectd3 associates with and polyubiquitinates Stat3, but not RORγt in effector CD4^+^ T cells of EAE mice.

### Hectd3 promotes non-degradative polyubiquitination of Stat3

**Fig. 8:**
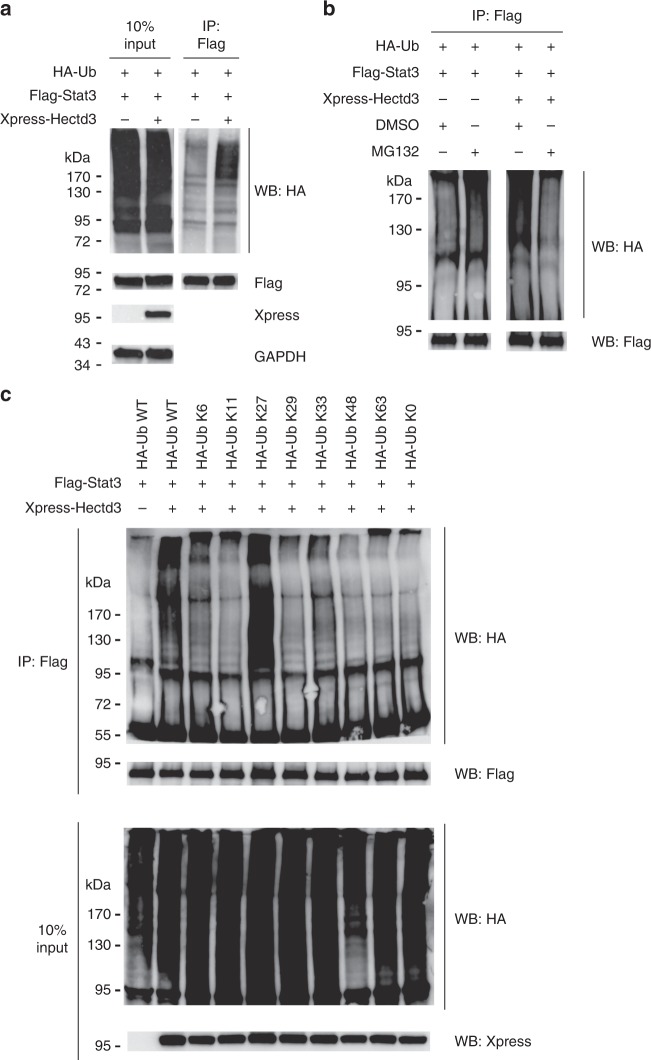
Hectd3 promotes non-degradative K27-linked polyubiquitin chains on Stat3. **a** Immunoblot of HA, Flag, Xpress, and GAPDH following Flag immunoprecipitation of protein extracts from HEK293T cells co-transfected as indicated with HA-Ub, Flag-Stat3, and Xpress-Hectd3. **b** Immunoblot of HA and Flag following immunoprecipitation of protein extracts from HEK293T cells co-transfected as indicated with HA-Ub, Flag-Stat3, and Xpress-Hectd3 vectors and treated for 4 h with 20 μM MG132 prior to protein extraction. **c** Immunoblot of HA and Flag following two-step Flag immunoprecipitation of protein extracts from HEK293T cells co-transfected with the indicated HA-Ub K only mutants, Flag-Stat3, and Xpress-Hectd3. HA-Ub K only mutants denote that the only lysine in the HA-tagged ubiquitin is at the indicated residue, and all other lysine residues are mutated to arginine. HA-Ub K0 mutant has all seven lysine residues mutated to arginine. **a**–**c** Immunoblots are representative of three independent experiments. Source data are provided as a Source Data file

We further characterized the Hectd3-mediated polyubiquitination of Stat3. Overexpression of Hectd3 increased polyubiquitination of transfected Stat3 without causing changes in its level under conditions of no treatment with proteasome inhibitors (Fig. 8a). The treatment with the proteasome inhibitor MG132 did not cause an increase in Stat3 polyubiquitination or its levels in the same conditions (Fig. 8b and Supplementary Fig. 7). These results suggest that Hectd3-mediated polyubiquitination of Stat3 does not lead to proteasomal degradation. MCL-1, known to be triggered for proteasomal degradation^47^, increased (Supplementary Fig. 7), demonstrating the efficiency of MG132 treatment. We further treated the cells with the translational inhibitor cycloheximide, which also did not impact Stat3 levels, but reduced MCL-1 levels, which is known to have a high rate of translation (Supplementary Fig. 7). Thus these results demonstrate that Hectd3 mediates a type of polyubiquitination that does not target Stat3 for proteasomal degradation and this is not masked by increased rates of Stat3 translation in the presence of Hectd3.

### Hectd3 promotes ubiquitination of Stat3 via K27 chains

We next sought to determine the type of polyubiquitin chains that Hectd3 promotes on Stat3. Using transfection of lysine (K) only ubiquitin mutants and two-step immunoprecipitation of Flag-tagged Stat3, we observed that the HA-Ub K27 only (HA-Ub K27) mutant was sufficient to promote ubiquitination of Stat3 in the presence of Hectd3 at the same level as WT HA-Ub (Fig. 8c). Conversely, we observed that Hectd3-mediated polyubiquitination was abolished in the HA-Ub-K27R mutant but not the WT HA-Ub or in HA-Ub-K11R, HA-Ub-K29R, HA-Ub-K48R, and HA-Ub-K63R mutants (Supplementary Fig. 8). Collectively, these results show that Hectd3 catalyzes non-degradative ubiquitination of Stat3 through K27-linked polyubiquitin chains.

### Stat3 linker and Hectd3 DOC domains mediate the interaction

**Fig. 9:**
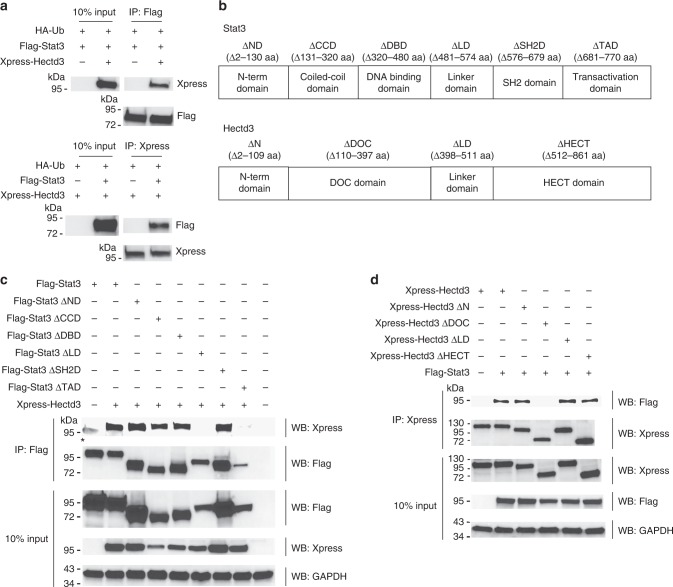
Stat3 linker region and Hectd3 DOC domain mediate the interaction between Stat3 and Hectd3. **a** Immunoblot of Xpress and Flag following Flag or Xpress immunoprecipitation of protein extracts from HEK293T cells co-transfected as indicated with HA-Ub, Flag-Stat3, and Xpress-Hectd3; data are representative of three independent experiments. **b** Stat3 and Hectd3 domains representation. **c** Immunoblot of Xpress, Flag, and GAPDH in the Flag-immunoprecipitate or input of protein extracts from HEK293T cells co-transfected as indicated with Flag-Stat3, Flag-Stat3 ΔND, Flag-Stat3 ΔCCD, Flag-Stat3 ΔDBD, Flag-Stat3 ΔLD, Flag-Stat3 ΔSH2D, or Flag-Stat3 ΔTAD, and Xpress-Hectd3; data are representative of two independent experiments. * denotes non-specific bands. **d** Immunoblot of Flag, Xpress, and GAPDH in the Xpress-immunoprecipitate or input of protein extracts from HEK293T cells co-transfected as indicated, with Xpress-Hectd3, Xpress-Hectd3 ΔND, Xpress-Hectd3 ΔDOC, Xpress-Hectd3 ΔLD, or Xpress-Hectd3 ΔHECT, and Flag-Stat3; data are representative of two independent experiments. Source data are provided as a Source Data file

We found that Hectd3 associates with Stat3 in CD4^+^ T cells and further show that they interact in transfected HEK293T cells as well (Fig. 9a). In addition, Hectd3 peptide sequences were detected in an unbiased manner by mass spectrometry in Stat3 complexes (Supplementary Tables 1, 2). To determine the domains of interaction between Stat3 and Hectd3, we generated Stat3 and Hectd3 domain truncation mutants (Fig. 9b). Removal of the Stat3 linker region, connecting the Stat3 DNA binding and SH2 domains, abolished the association with Hectd3 (Fig. 9c). Vice versa, deletion of the Hectd3 DOC domain (amino acids 110-397) resulted in inability of Hectd3 to associate with Stat3 (Fig. 9d). Thus, these data demonstrate that the Stat3 linker region interacts with the Hectd3 DOC domain to promote the association between the two proteins.

### Stat3 K180 ubiquitination by Hectd3 in Th17 cell generation

**Fig. 10:**
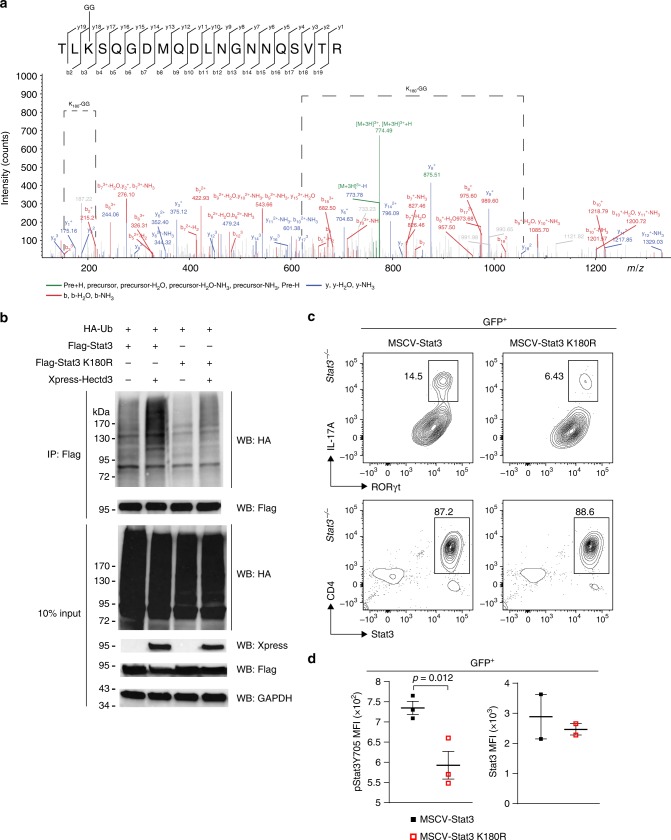
Ubiquitination of Stat3 at K180 by Hectd3 is essential for RORγt^+^IL-17A^hi^ Th17 cell generation. **a** HEK293T cells were co-transfected with HA-Ub, Flag-Stat3, and Xpress-Hectd3. Extracts were immunoprecipitated with anti-Flag antibodies, followed by trypsin digestion and tandem mass spectrometry, as described in Material and methods. A fragmentation spectrum of ubiquitinated TLkSQGDMQDLNGNNQSVTR peptide (ubiquitinated K180 residue) of Stat3. Parent ion corresponding to TLkSQGDMQDLNGNNQSVTR peptide mass (774.0342, *z* = +3, retention time *t* = 38.1356 min) has been subjected to higher-energy collisional dissociation in mass spectrometer. The detected b- and y-fragment ion series have been annotated and mass difference corresponding to GG tag (114.04293 Da) has been assigned to a K3 residue as indicated (K180-GG) by the difference between the y17 and y18 fragment ion masses. **b** Representative immunoblot of HA, Flag, Xpress, and GAPDH following Flag immunoprecipitation of protein extracts from HEK293T cells co-transfected as indicated with HA-Ub, Flag-Stat3, Flag-Stat3 K180R, and Xpress-Hectd3; data are representative of three independent experiments. **c** Flow cytometry analysis of intracellular IL-17A, and intranuclear RORγt and Stat3 in GFP^+^ *Stat3*^−/−^ CD4^+^ T cells transduced with MSCV-Stat3 or MSCV-Stat3 K180R retroviruses and in vitro polarized under Th17 conditions. Representative of three independent experiments. Gating strategy was first on GFP^+^ T cells. **d** MFI of pStat3 Y705 and Stat3 in GFP^+^ *Stat3*^−/−^CD4^+^ T cells transduced with indicated retroviruses normalized to those transduced with MSCV empty vector and in vitro polarized under Th17 conditions. Data (*n* = 6) are mean of three (pStat3 Y705) and two (Stat3) independent experiments and are presented as mean ± SEM; *p* value was obtained from Student’s *t* test. Source data are provided as a Source Data file

We further conducted tandem mass spectrometry analysis of Flag-Stat3 complex from HEK293T cells co-transfected with HA-Ub, Flag-Stat3, and with or without Xpress-Hectd3. Stat3 K180 was identified as having the K-ε-GG residue only in HEK293T cells transfected with Hectd3 (Fig. 10a and Supplementary Table 3). Site-directed mutagenesis was performed to generate a Flag-Stat3 K180R mutant. Stat3 polyubiquitination was abolished in this mutant, demonstrating that Stat3 K180 is a target site for Hectd3-mediated polyubiquitination (Fig. 10b) and *Stat3*^−/−^ CD4^+^ T cells transduced with the Stat3 K180R mutant retroviruses showed a reduction in the generation of RORγt^+^IL17^hi^ population in Th17-polarizing conditions, compared to those transduced with WT Stat3 retroviruses (Fig. 10c). Moreover, the level of pStat3 Y705 was reduced in *Stat3*^−/−^ CD4^+^ T cells transduced with the Stat3 K180R mutant retrovirus compared to those transduced with WT Stat3 retroviruses, suggesting that Stat3 K180 is important in promoting phosphorylation of Stat3 at Y705 in Th17 cells (Fig. 10d). The expression level of Stat3 and Stat3 K180R in transduced *Stat3*^*−/−*^ CD4^+^ Th17 cells was equal (Fig. 10c, d), which suggests K180 does not play a role in degradation of Stat3 in Th17 cells. These results show that Hectd3-mediated ubiquitination of Stat3 at K180 promotes Stat3 activating phosphorylation at Y705 and generation of RORγt^+^IL17^hi^ Th17 cells.

## Discussion

In this study, we demonstrate that Hectd3 ubiquitinates Malt1 and Stat3 in a non-degradative manner to promote differentiation of pathogenic Th17 cells in EAE. In contrast to Cbl-b, Itch, and GRAIL, which are E3 ubiquitin ligases induced following T cell activation^48^, we found that the expression of Hectd3 is constitutive in naïve as well as in activated, polarized CD4^+^ T cells, and is not needed for T cell activation or in vitro polarization to Th1, Th2, and Treg cells. However, Hectd3 is required for polarization in Th17 conditions. In line with this, EAE severity was attenuated in *Hectd3*^−/−^ mice, and *Hectd3*^−/−^ CD4^+^ T cells produced decreased amounts of IL-17A and GM-CSF, had lower levels of RORγt, and poorly infiltrated the CNS. Polyubiquitination of Malt1 and Stat3 was diminished in *Hectd3*^−/−^ CD4^+^ T cells from mice with EAE, which suggests that Hectd3 controls Th17 cell function through polyubiquitination of these two targets. Using ubiquitin mutants, we found that Hectd3 promotes K27- and K29-linked polyubiquitin chains on Malt1A, and K27-linked polyubiquitin chains on Stat3. Further experiments employing tandem mass spectrometry analysis and retroviral reconstitution revealed that Hectd3 targets Malt1A K648 and Stat3 K180 residues for non-degradative polyubiquitination, which are essential for the generation of RORγt^+^IL-17A^hi^ Th17 cells.

Previously it was shown that monoubiquitination of Malt1 at K644 was sufficient and essential for Malt1 protease activity^42^. Malt1 was shown to bind Bcl10 through Malt1 N-terminal DD, without the need of the C-terminus^49^, thus decreasing the likelihood that Malt1A K648, identified by us as a target for Hectd3 ubiquitination, has a direct impact on CBM complex formation. In agreement with this, our results show that Hectd3-dependent Malt1A K648 polyubiquitination did not affect Malt1 substrate cleavage and CBM complex formation in Jurkat cells. Malt1 was also shown to be ubiquitinated by TRAF6 at six C-terminal lysine residues following TCR stimulation, which was required for NF-κB activation^41^. We show here that Hectd3-mediated polyubiquitination of Malt1 solely at K648 affected p65 nuclear translocation in Th17 cells, which suggests a yet undiscovered mechanism underlying Malt1 control of NF-κB activation that is regulated by Malt1 K648. Given that RelA/p65 was shown to control *Rorc* expression^50^, Hectd3-mediated polyubiquitination of Malt1A K648 may contribute to transcriptional regulation of *Rorc* by p65 and optimal IL-17A production, but how polybubiquitination of Malt1A K648 promotes p65 nuclear translocation has yet to be elucidated.

Hectd3 did not promote RORγt polyubiquitination, but exerted control over RORγt level through regulating Stat3 activating phosphorylation at Y705, and thus promoting Th17 pathogenicity. We found Stat3 as a target of Hectd3 for non-degradative polyubiquitination in agreement with previous studies showing that Hectd3 mediates types of polyubiquitination not associated with proteasomal degradation but with signaling, controlling substrate activity on caspase-8^31^, caspase-9^32^, Malt1^30^ (and this study), and TRAF3^33^. We found that Hectd3 mediates K27-linked polyubiquitin chains on Stat3 and identified K180, within the Stat3 coiled-coil domain, as the target for polyubiquitination, which was important for proper Stat3 Y705 phosphorylation and generation of RORγt^+^IL-17A^hi^ Th17 cells. One study found that TRAF6-mediated ubiquitination of Stat3 at six lysine residues in the SH2 domain promoted phosphorylation of Stat3 Y705 in mouse embryonic fibroblasts infected with *Salmonella* *typhimurium*^51^; however, the in vivo significance of this TRAF6-mediated ubiquitination on Stat3 activation remains to be determined. We showed that Stat3 uses its linker region, located between DNA binding domain and SH2 domain, to associate with the Hectd3 DOC domain.

The regulation of Stat3 Y705 phosphorylation and activation by Hectd3-mediated polyubiquitination is similar to the regulation of PLCγ-1 phosphorylation by Cbl-b-mediated ubiquitination. In both cases, the cross talk between ubiquitination and phosphorylation is not associated with proteasomal degradation. However, whereas Hectd3-mediated polyubiquitination of Stat3 promotes phosphorylation and activation of Stat3, Cbl-b ubiquitination of PLCγ-1 inhibits phosphorylation and activation of PLCγ-1^52^. Cross talk between ubiquitination and phosphorylation is well characterized in antiviral innate immunity^53^, but is limited to proximal TCR signaling and CBM complex signal transduction in T cell biology^54^. We present a mechanism wherein pStat3 Y705 is reduced in the absence of Hectd3 in vivo during EAE, as well as in Stat3 K180R mutant in Th17 cells, thus linking ubiquitination by an E3 ubiquitin ligase to phosphorylation and activation of a critical Th17 transcription factor. Hectd3-Stat3 ubiquitination-phosphorylation interplay may be similar to the ubiquitination of IRF7 by TRAF6 in promoting phosphorylation and activation of IRF7 in innate antiviral pathways^55^. Stat3 polyubiquitination by Hectd3 at K180 may promote pStat3 Y705 through recruitment of a kinase, protection from a phosphatase, or a novel mechanism.

Ubiquitination and deubiquitination have been shown to play crucial roles in regulation of T cell immunity^54^. The deubiquitinase DUBA and the E3 UBR5 were shown to negatively regulate Th17 response. Specifically, DUBA deubiquitinates and stabilizes UBR5, then UBR5 ubiquitinates and targets RORγt for proteasomal degradation^23^. The deubiquitinases USP4^26^ and USP17^28^ stabilize RORγt level through deubiquitination, preventing its proteasomal degradation. Itch was also shown to polyubiquitinate and target RORγt for proteasomal degradation, thus inhibiting colitis-associated colorectal cancer and colon inflammation^21^. USP15 was found to deubiquitinate RORγt at K446 to promote co-activator recruitment^27^. The E3 PDLIM2 was found to induce polyubiquitination and proteasomal degradation of Stat3, inhibiting the Th17 granulomatous response^24^. A theme emerges from these studies, namely, ubiquitination negatively impacts Th17 function. In contrast, we have demonstrated a positive regulation of Th17 function by the E3 ubiquitin ligase Hectd3. Moreover, Hectd3-mediated polyubiquitination of Stat3 at K180 does not target Stat3 for proteasomal degradation, but rather promotes the activating Y705 phosphorylation to enhance RORγt and IL-23R expression, as well as IL-17A and GM-CSF production. Similarly, polyubiquitination of Malt1 by Hectd3 at K648 does not promote Malt1 degradation, but positively regulates generation of RORγt^+^IL17^hi^ Th17 cells.

We have characterized that Hectd3 mediates K27- and K29-linked polyubiquitin chains on Malt1 and K27-linked polyubiquitin chains on Stat3. Previous studies have shown that Hectd3 mediates K63-linked and K27/29-linked polyubiquitin chains on caspase 8^31^ and caspase 9^32^, respectively. K27-linked polyubiquitination has been shown to be involved in signaling-complex assembly in T cells^56^ and innate immunity^57^, and K29-linked polyubiquitin chains mediated by TRIM13 on TRAF6 promotes TLR2 signaling in macrophages^58^. Along the same line, Hectd3-mediated K27- and K29-linked polyubiquitin chains on Malt1 and K27-linked polyubiquitin chains on Stat3 play a role in promoting Malt1 and Stat3 signaling, respectively. It is known that the C-lobe of HECT domain controls linkage specificity and that linkage-specific chain formation is dependent on ubiquitin residues adjacent to particular acceptor lysines^59,60^. Hectd3 was found to bind both Malt1^30^ and Stat3 through its DOC domain. Malt1 interacts with Hectd3 through N-terminal DD^30^ and Stat3 interacts with Hectd3 through a linker domain, between the DNA-binding domain and SH2 domain. The targeted lysine by Hectd3 in Malt1, K648, is at the C-terminus, while in Stat3, K180, is located in the coiled-coil domain. Steric hindrance between Hectd3 domains and Malt1, Stat3, and other substrate domains may play a role in determining the linkage specificity of Hectd3-mediated polyubiquitination on a particular substrate. Malt1 also uses DD to associate with BCL10 in the formation of CBM complex^49^, thus raising the possibility that Hectd3 competes with BCL10 for Malt1 DD, and this competition may affect Hectd3 linkage specificity.

In the context of Th17 cells, the Malt1 and Stat3 pathways have not been previously shown to intersect. We have identified a common post-translational mechanism, namely, non-degradative ubiquitination mediated by Hectd3, that controls both Malt1 and Stat3 pathways in Th17 cells. Abolition of polyubiquitination at Malt1A K648 or Stat3 K180 was independently sufficient to reduce IL-17A production by Th17 cells, which suggests that Hectd3-mediated polybubiquitination of Malt1 and Stat3 are independent from each other and both are non-redundant in the generation of RORγt^+^IL-17A^hi^ cells. It is unknown if Malt1 and Stat3 are in a common ubiquitination signalosome with Hectd3, but mass spectrometry analysis of Flag-Malt1A and Flag-Stat3 in HEK293T cells did not yield Stat3 peptides in the Flag-Malt1A, or Malt1 peptides in the Flag-Stat3 immunoprecipitates. A recent mouse lung cancer model study found that Malt1 controls Stat3 activation in vivo downstream of EGFR through NF-κB-dependent autocrine/paracrine IL-6 production^61^, raising the possibility that the Malt1 pathway may intersect the Stat3 pathway in Th17 cells. Exploiting the ubiquitination system for therapeutic intervention is gaining prominence due to the specificity conferred by E3 ubiquitin ligases and the benefit of modulating molecular switches in favoring beneficial outcome^62^, as well as the possibility of designing selective E3 inhibitors^63^. Therefore, Hectd3 may be a favorable target for MS, other Th17-related diseases such as psoriasis, or cancer therapy since Stat3 activation is instrumental in their pathogenesis.

## Methods

### Mice

Mice with a targeted deletion of *Hectd3* on a segregating 129/SvEv-C57BL/6 background were obtained from Taconic Biosciences (Rensselaer, NY, USA) (Cat#TF2706), and backcrossed to C57BL/6NTac (Taconic Biosciences) for at least ten generations (*Hectd3*^*−/−*^). All experiments were performed using 10–20-week-old male and female mice. However, only female mice were used for experiments requiring EAE disease scoring^64^. *Hectd3*^−/−^ mice are born in normal Mendelian ratios, retain fecundity, and are healthy. *Malt1*^*−/−*^ mice were obtained from Dr. Robert Rickert (Sanford Burnham Prebys Medical Discovery Institute, La Jolla, CA, USA) with the approval of Genentech (South San Francisco, CA, USA)^65^. B6.129S1-Stat3^tm1Xyfu^/J (Stat3^flox^) mice were purchased from Jackson Laboratory (Bar Harbor, ME, USA). *Stat3*^*f/f*^ mice were crossed with Tg(Cd4-cre)1Cwi/BfluJ(CD4-cre) mice (Jackson Laboratory, Bar Harbor, ME, USA) to generate Stat3 conditional knockout mice. All mice were housed under SPF conditions in the Animal Care Service facilities of the University of Florida College of Medicine (UFCOM), on 12-h light and 12-h dark cycles and 25±2 °C. All protocols were approved by the University of Florida Institutional Animal Care and Use Committee (UF IACUC).

### Mouse cell purification

Primary mouse CD4^+^ T cells (Cat#19852, STEMCELL Technologies, Vancouver, Canada) from dLNs during EAE or CD4^+^ T (Cat#19852, STEMCELL Technologies, Vancouver, Canada), CD8^+^ T (Cat#19853, STEMCELL Technologies, Vancouver, Canada), and CD11b^+^ myeloid (Cat#18970, STEMCELL Technologies, Vancouver, Canada) cells from spleen and peripheral lymph nodes at steady state were purified following manufacturer’s instructions, after mashing through a 40-μm nylon filter (Cat#22363547, Thermo Fisher Scientific, PA, USA). Isolated CD4^+^ T cells were stimulated at 37 °C and 5% CO_2_ for 30 min in 10% RPMI containing PMA (20 ng/ml) and Ionomycin (1 µg/ml) for Malt1 and Stat3 ubiquitination analysis.

### In vitro polarization of CD4^+^ T cells

CD4^+^ T cells were purified from steady state *Hectd3*^−/−^, *Malt1*^−/−^, and WT mice as above. Anti-CD3 (Cat#BE0002, Bio X Cell, NH, USA) was diluted in PBS to 5 μg/ml and plated on flat-bottom 96-well plates (Cat#353072, Corning, NY, USA) at 4 °C overnight to bind anti-CD3 to plate. Purified CD4^+^ T cells at 2 × 10^6^ cells/ml were cultured in wells of anti-CD3 plates with the following antibodies and cytokines for each T helper subset polarizing condition in 10% RPMI: Th0, 2 μg/ml anti-CD28 (Cat#BE0015-1, Bio X Cell, NH, USA), 100 U/ml IL-2 (Cat#100-12, Shenandoah Biotechnology, PA, USA); Th1, 2 μg/ml anti-CD28, 100 U/ml IL-2, 10 μg/ml anti-IL-4 (Cat#BE0045, Bio X Cell, NH, USA), 10 ng/ml IL-12 (Cat#200-25, Shenandoah Biotechnology, PA, USA); Th2, 2 μg/ml anti-CD28, 100 U/ml IL-2, 10 μg/ml anti-IFNγ (Cat#BE0055, Bio X Cell, NH, USA), 10 μg/ml anti-IL-12 (Cat#BE0052, Bio X Cell, NH, USA), 10 ng/ml IL-4 (Cat#200-18, Shenandoah Biotechnology, PA, USA); Th17, 2 μg/ml anti-CD28, 10 μg/ml anti-IFNγ, 10 μg/ml anti-IL-12, 10 μg/ml anti-IL-4 (Cat#BE0045, Bio X Cell, NH, USA), 5 ng/ml TGF-β3 (Cat#800-07, Shenandoah Biotechnology, PA, USA), 10 ng/ml IL-6 (Cat#200-02, Shenandoah Biotechnology, PA, USA), 10 ng/ml IL-1β (Cat#200-68, Shenandoah Biotechnology, PA, USA), 20 ng/ml IL-21 (Cat#200-70, Shenandoah Biotechnology, PA, USA), 50 ng/ml IL-23 (Cat#589004, Biolegend, CA, USA); Treg, 2 μg/ml anti-CD28, 100 U/ml IL-2, 10 μg/ml anti-IFNγ, 20 ng/ml TGF-β1 (Cat#100-39, Shenandoah Biotechnology, PA, USA). Cells were polarized at 37 °C, 5% CO_2_ for 3 days, then fresh medium with polarizing cytokines and antibodies was added, and cells were polarized at 37 °C, 5% CO_2_ for 3 additional days. In vitro polarized CD4^+^ T cells were then stimulated with 20 ng/ml PMA and 1 µg/ml ionomycin for 4 h with 10 µg/ml Brefeldin A added 1 h into the 4-h stimulation. Following PMA/ionomycin stimulation, cells were surface stained, permeabilized, then stained for intranuclear transcription factors and intracellular cytokines^66^.

### Active EAE Induction

MOG_35–55_ peptide with amino acid sequence MEVGWYRSPFSRVVHLYRNGK was purchased from Mimotopes (Mimotopes, Victoria, Australia) and resuspended in 0.09% saline solution at a concentration of 2 mg/ml. Fresh complete Freund’s adjuvant (CFA) was reconstituted with 4 mg/ml non-viable, desiccated *Mycobacterium tuberculosis* H37 Ra (Cat#231141, BD, MD, USA) in incomplete Freund’s adjuvant (Cat#F5506, Sigma, Millipore-Sigma, MO, USA). Pertussis toxin from *Bordetella pertussis* in glycerol (Cat#179B, List Biological Laboratories, CA, USA) was freshly diluted in PBS to 3 μg/ml. MOG_35–55_/CFA emulsion was freshly reconstituted by mixing equal volume of 2 mg/ml MOG_35–55_ with CFA and vortexed for 30 min at room temperature. For each mouse, a total of 200 μl MOG_35–55_/CFA emulsion was subcutaneously injected at flank and scruff sites; 100 μl of 3 μg/ml pertussis toxin was administered intraperitoneally 1 h and 24 h following MOG_35–55_/CFA emulsion. Clinical scoring was performed starting 7 days following active EAE induction, every day until the completion of the experiment. Clinical scoring was established as follows: score 0.5: weakness of tail; score 1: flaccid tail; score 1.5: flaccid tail and hind limb inhibition marked by hind limbs falling through cage bars; score 2: flaccid tail, weak hind limbs marked by hind limbs held close together when picked up at the base of the tail; score 2.5: flaccid tail, both hind limbs are dragging when moving or complete paralysis of one hind limb but not the other; score 3: flaccid tail, hind limb paraparesis; score 3.5: flaccid tail, hind limb paraparesis and one forelimb paralysis; score 4: flaccid tail, quadriplegia; score 5: moribund, euthanasia.

### Passive induction of EAE

Donor mice were induced with active EAE as described above, but without pertussis toxin. Eleven days after active EAE induction, dLN cells were collected, RBC lysed, and incubated in 10% RPMI containing 10% FBS (Cat#SZ-0501, Hooke Laboratories Inc., MA, USA) with 20 µg/ml MOG_35–55_ peptide (Mimotopes, Victoria, Australia), 10 µg/ml anti-mouse IFNγ (Cat#BE0055, Bio X Cell, NH, USA), 20 ng/ml recombinant mouse IL-23 (Cat#589004, Biolegend, CA, USA) at 3 million cells/ml for 72 h at 37 °C, 5% CO_2_, for in vitro reactivation. In vitro reactivated mouse CD4^+^ T cells were purified as above, and 20 million in vitro reactivated CD4^+^ T cells were transferred into recipient mice intraperitoneally. Clinical scoring of recipient mice was performed starting 5 days following adoptive/passive transfer of in vitro reactivated CD4^+^ T cells, every day until the completion of the experiment.

### Isolation of leukocytes from brain and spinal cord

Leukocytes were isolated from brain and spinal cord^66^. Mice were euthanized with CO_2_, hepatic portal vein was severed, and left ventricle of heart was perfused with at least 10 ml of ice-cold PBS. Perfused brain and spinal cord were dissected out and placed in gentleMACS C tubes (Cat#130-093-237, Miltenyi Biotec Inc., CA, USA) containing 3 ml of RPMI-1640 supplemented with 1% l-glutamine, 1% non-essential amino acids, 1% sodium pyruvate, 1% penicillin/streptomycin, 0.01 M HEPES, 220 μM 2-mercaptoethanol, and 1% FBS (1% RPMI). Brain and spinal cord tissue were then dissociated using gentle MACS Dissociator (Cat#130-093-235, Miltenyi Biotec Inc., CA, USA) setting m_brain_03.01, m_brain_02_02, and m_brain_01_02 for C tube. Dissociated brain and spinal cord were pressed through 70-μm nylon mesh (Cat#22363548, Thermo Fisher Scientific, PA, USA) with 1% RPMI up to a volume of 7 ml total; 100% isotonic Percoll was prepared by mixing 46.25 ml Percoll (Cat#17-0891-01, GE Healthcare Bio-Sciences, PA, USA) with 3.6 ml 10× HBSS (Cat#20-023-CV, Corning, NY, USA), and 0.6 ml 7.5% sodium bicarbonate. 100% isotonic Percoll was diluted to 70% isotonic Percoll with 1% RPMI; 3 ml of 100% isotonic Percoll was added to the 7 ml brain and spinal cord homogenate for a final 30% Percoll brain and spinal cord homogenate. The 10 ml 30% Percoll brain and spinal cord homogenate was then underlaid with 2 ml 70% isotonic Percoll using a glass Pasteur pipette. The 30–70% layered Percoll was centrifuged for 30 min, 500 × *g* at room temperature without interruption. The 30/70% interphase was collected with a transfer pipette following centrifugation, and washed with at least threefold volume of 1% RPMI, then centrifuged for 10 min at 350×*g*, 4 °C for collecting leukocytes.

### Stimulation of cells for intracellular cytokine staining

For detection of cytokines by intracellular flow cytometry, cells were cultured at 37 °C and 5% CO_2_ for 4 h in IMDM media containing PMA (20 ng/ml) and Ionomycin (1 µg/ml). Brefeldin A (10 µg/ml) was added after 1 h. After incubation, the cells were washed and stained with Fixable Viability Dye (Affymetrix) and surface markers. Cells were then fixed and permeabilized with Foxp3 Fix/Perm kit (Affymetrix, #00-5523-00), then subsequently stained for cytokines.

### Flow cytometry

Flow cytometry was performed on a BD LSR II, upgraded by Cytek Biosciences, with data acquired using BD FACS DIVA software. All data were analyzed using FlowJo (TreeStar).

### Flow cytometry antibodies

Cells were stained with the following antibodies: 0.1 µl/100 μl Fixable Viability Dye (eFluor 520, eFluor 780), 0.25 µg/100 μl CD4 (Brilliant violet 711, clone: GK1.5), 0.5 µg/100 μl RORγt (APC, clone: AFKJS-9), 0.4 µg/100 μl T-bet (PE-cyanine7, clone: 4B10), 0.5 µg/100 μl IL-17A (eFluor 450, clone: eBio17B7), 0.25 µg/100 μl GM-CSF (PE, clone: MP1-22E9), 0.25 µg/100 μl IFNγ (FITC, clone: XMG1.2), 0.5 µg/100 μl CD25 (PE-Cy7, clone: PC61), 0.06 µg/100 μl Foxp3 (eFluor 450, FITC, clone: FJK-16s), 0.5 µg/100 μl CD8α (PE, clone: 53-6.7), 5 µg/100 μl IL-23R (PE, BV711, clone: O78-1208), 0.5 µg/100 μl CCR6 (BV605, clone: 29-2L17), 1 µg/100 μl CD29 (PE-Cy7, clone: HMβ1-1), 2 µl/100 μl Malt1 (#2494 Cell Signaling Technology, Danvers, MA, USA), 5 µg/ml Alexa Fluor 488 goat anti-rabbit IgG (H + L) (A11008, Fisher Scientific, Thermo Fisher Scientific, PA, USA), 2 µl/100 μl pStat3 Y705 (#4324, Alexa Fluor 647, clone: D3A7), 0.25 µg/100 μl CD44 (APC, clone: IM7), 0.5 µg/100 μl CD62L (PE, clone: MEL-14), 0.5 µg/100 μl CD69 (PerCP-Cy5.5, clone: H1.2F3), 0.25 µg/100 μl Ki-67 (FITC, clone: SolA15), 1 µl/100 μl NF-κB p65 (#4764, clone: C22B4), 1 µl/100 μl RelB (#4922, clone: C1E4), 5 µg/ml GFP (A21311, Alexa Fluor 488), 0.125 µg/100 μl CD90.1 (PE-Cy7, clone: HIS51).

### Fluorescence activated cell sorting

Spleen and lymph nodes were pressed through 40-μm nylon mesh (Cat#22363547, Thermo Fisher Scientific, PA, USA). Cells were surface stained with anti-B220 antibodies (FITC, clone: RA3-6B2) and sorted using BD FACSAriaIII (BD Biosciences, CA, USA).

### Imaging flow cytometry

CD4^+^ T cells isolated from WT or *Hectd3*^*−/−*^ mice induced with EAE were stimulated with PMA (20 ng/ml) and Ionomycin (1 µg/ml) for 60 min. Following stimulation, cells were collected in 0.1% BSA/PBS, centrifuged at 4 °C, 400 × *g*, 8 min, then blocked with 0.1% BSA/PBS/2.4G2. Cells were stained with surface CD4 and intracellular p65, RelB, and DAPI markers. Cells were stained with CD4 for 20 min on ice, then washed twice with 0.1% BSA/PBS. Cells were then fixed with 1% PFA for 30 min at room temperature, then washed with 0.1% BSA/PBS, collected at 500 × *g* for 5 min at 4 °C. Cells were permeabilized with 0.1% Triton X-100 in PBS for 5 min at room temperature, collected at 500 × *g* for 5 min at 4 °C, then blocked with 0.1% Triton X-100 in PBS with 2.4G2 blocking buffer. This was followed by intracellular staining with anti-p65 (1:100) or -RelB (1:100) antibodies, followed by incubation at room temperature for 25 min. Cells were washed with 0.1% BSA/PBS, then stained with Alexa Fluor 488 goat anti-rabbit IgG (H + L) (1:1000) for 20 min. at room temperature. Cells were washed with 0.1% BSA/PBS, then fixed with 1% PFA/PBS for 10 min at room temperature and collected at 500 × *g* for 5 min at 4 °C. Cells were resuspended in 1% PFA/PBS with 0.2 µg/ml DAPI and incubated on ice for 5 min, followed by image acquisition immediately. Imaging flow cytometry was performed using an Amnis ImageStreamX MKII imaging flow cytometer and data were analyzed using IDEAS 6.1 software (Amnis).

### Cells

HEK293T cells (Cat#CRL-3216, ATCC, VA, USA), were grown in DMEM supplemented with 1% l-glutamine, 1% non-essential amino acids, 1% sodium pyruvate, 1% penicillin/streptomycin, 0.01 M HEPES, 55 μM 2-mercaptoethanol, and 10% FBS (10% DMEM). Platinum-E (Plat-E) retroviral packaging cells (Cat#RV-101, Cell Biolabs, CA, USA) were grown in 10% DMEM. EL4 cells (Cat# TIB-39, ATCC, VA, USA) or male and female primary CD4^+^ T cells, isolated as above, were cultured in RPMI-1640 supplemented with 1% l-glutamine, 1% non-essential amino acids, 1% sodium pyruvate, 1% penicillin/streptomycin, 0.01 M HEPES, 220 μM 2-mercaptoethanol, and 10% FBS (10% RPMI). Generation of MALT1KO Jurkat cells was performed as described^40^. All mammalian cell cultures were grown at 37 °C and 5% CO_2_. *Escherichia coli* strain MAX Efficiency DH5α competent cells (Cat#18258012, Invitrogen, Thermo Fisher Scientific, PA, USA) were used for plasmid transformation and preparation. Transformed *E. coli* DH5α were grown at 37 °C and 230 rpm shaking.

### Vector constructs

HA-Ub vector was a generous gift from Dr. Carlos M. de Noronha. Mouse Hectd3 was cloned into pcDNA3.1/His C mammalian expression vector (Cat# V38520, Invitrogen, Thermo Fisher Scientific, PA, USA). PCR product was obtained using Premix Taq DNA Polymerase (Ex Taq Version 2.0) according to manufacturer’s protocol (Cat#RR003A, Takara Bio USA, CA, USA) with the Hectd3 primers listed in Supplementary Table 4, and template mouse Hectd3 clone (Cat#MC206119, Origene, MD, USA). Hectd3 PCR product containing 5′ BglII site and 3′ XhoI site were subcloned into pGEM-T vector following manufacturer’s protocol (Cat#A3600, Promega, WI, USA). Subcloned Hectd3 pGEM-T were digested with BglII and XhoI, while pcDNA3.1/HisC was digested with BamHI and XhoI restriction enzymes. Digested Hectd3 cDNA clone and digested pcDNA3.1/HisC were ligated using Quick Ligation Kit following manufacturer’s instructions (Cat#M2200, New England Biolabs, MA, USA). Vector construct pCMV6-Flag-Stat3 (Cat#MR227265) is from Origene, MD, USA. pEF 3xFlag-MALT1A (Flag-Malt1A) and MSCV-3xFlag-MALT1A-IRES-Thy1.1 (MSCV-Malt1A) vectors were previously described^40^. Mouse Stat3 was cloned into MSCV (Supplementary Table 4) with pCMV6-Flag-Stat3 and pCMV6-Flag-Stat3 K180R as templates. pVPack-VSV-G (Cat#217567) and pVPack-GP (Cat#217566) were purchased from Agilent Technologies, CA, USA. Vector constructs were prepared using ZymoPURE plasmid midiprep kit (Cat#D4201, Zymo Research, CA, USA) following manufacturer’s instructions.

### Site-directed mutagenesis for point mutation and truncation

All mutants were generated using Q5 site-directed mutagenesis kit (Cat#E0554S, New England Biolabs, MA, USA) following manufacturer’s protocol or standard cloning. All primers used are listed in Supplementary Table 4.

### Retrovirus production

Retroviral particles were produced as follows^67^. Plat E cells were seeded on 10-cm cell cultures plates such that 80% confluence was reached overnight. Media was aspirated off Plat E cell culture and 5 ml of fresh 10% DMEM was added; 9 μg MSCV-MALT1A or MSCV-Stat3 or 9 μg MSCV-MALT1A K648R or MSCV-Stat3 K180R vectors were added into serum-free DMEM along with 4.5 μg pVPack-VSV-G and 9 μg pVPack-GP to a total volume of 450 μl, then incubated at room temperature for 5 min. Lipofectamine 2000 (36 μl) was added to 414 μl of serum-free DMEM and incubated at room temperature for 5 min. The Lipofectamine solution was added to the DNA vectors tube, mixed by flicking, then incubated at room temperature for 20 min. The DNA-Lipofectamine 2000 complex with a total volume of 900 μl was added dropwise to Plat E cells. The transfected Plat E cells were incubated overnight at 37 °C, 5% CO_2_. The medium was aspirated from transfected Plat E cells; then 5.5 ml of pre-warmed (37 °C) 10% RPMI was added to the edge of the plate slowly, then the transfected Plat E cells in 10% RPMI were incubated overnight at 37 °C, 5% CO_2_. Retroviral particles in 10% RPMI media were collected and filtered through a 0.45-μm syringe filter (Cat#431220, Corning, NY, USA) and collected at 48 h and 72 h post-transfection. Retrovirus-containing 10% RPMI medium was stored at −80 °C until use for reconstitution assay.

### Retrovirus reconstitution assay

Primary mouse spleen and peripheral lymph node WT or *Malt1*^−/−^ or *Stat3*^*f/f*^ *CD4-cre* CD4^+^ T cells were isolated following manufacturer’s protocol (Cat#19852, STEMCELL Technologies, Vancouver, Canada). Primary mouse CD4^+^ T cells were transduced as follows^67–70^. Following activation with anti-CD3/anti-CD28 antibody-bounded beads (Cat#11452D, Invitrogen, Thermo Fisher Scientific, PA, USA) at 2 × 10^6^ cells/ml of 10% RPMI supplemented with 20 U/ml IL-2, and incubation at 37 °C, 5% CO_2_ overnight, cells were transduced on retronectin-coated wells. Retronectin (15 μg/ml; Cat#T100A, Takara Bio USA, CA, USA) in PBS was used to coat 24-well cell culture plates for 3 h at room temperature, just before transduction, and then aspirated and wells were washed with 0.5% FBS in PBS. Retrovirus-containing 10% RPMI (250 µl) was added to each well of a 24-well plate, and activated primary mouse CD4^+^ T cells were added to the retrovirus-containing medium immediately. The 24-well plate was then centrifuged at 2000 × *g* at 30 °C for 1 h, then incubated at 37 °C and 5% CO_2_ for 24 h. The supernatant (250 μl) was then carefully aspirated off each well, then 250 μl of freshly thawed retrovirus-containing 10% RPMI was added to each well. For a second time, the 24-well plate was then centrifuged at 2000 × *g* at 30 °C for 1 h, then incubated at 37 °C, 5% CO_2_ for an additional 24 h. The retrovirus-transduced CD4^+^ T cells were then subjected to in vitro Th17 polarizing conditions as described above. Th17 in vitro polarized retrovirus-transduced CD4^+^ T cells were then stimulated with 20 ng/ml PMA and 1 µg/ml ionomycin for 4 h with 10 µg/ml Brefeldin A added 1 h before harvesting cells. Cells were then surface stained, permeabilized, then stained for intranuclear transcription factors and intracellular cytokines.

### EL4 cells transduction

Retrovirus-containing 10% RPMI (250 µl) along with 8 µg/ml Polybrene was added to each well of a 24-well plate, and EL4 or MALT1KO Jurkat cells were added to the retrovirus-containing medium immediately.

### MALT1KO Jurkat cells transduction

Generation of MALT1-deficient Jurkat T cells has been described^40^. For reconstitution, cDNAs of MALT1 isoform A WT and mutant K648R linked to a C-terminal Flag-Strep-Strep tag were cloned into a pHAGE lentiviral expression vector in frame with hΔCD2 and the co-translational processing site T2A. For lentivirus production, HEK293T cells were transfected with 1.5 µg of psPAX2 (Addgene #12260; gift D. Trono), 1 µg of pMD2.G (Addgene #12259; gift D. Trono), and 2 µg of pHAGE transfer vector. Lentivirus particles containing MALT1 were collected 72 h after transfection, filtrated and added to MALT1KO Jurkat T cells in the presence of polybrene (8 µg/ml). After 24 h, the medium containing viral particles was replaced by viral particle-free RPMI medium. Culture of cells for 1 week revealed >95% ΔCD2-positive cells by FACS analysis using an Attune Flow Cytometer (Applied Biosystems).

### Transfection

Transfection of HEK293T cells was performed using Lipofectamine 2000 (Cat#11668019, Invitrogen, Thermo Fisher Scientific, PA, USA) according to manufacturer’s protocol. HEK293T cells were seeded on either 6-well or 10-cm plates overnight at 70% confluence. Transfection of HEK293T cells seeded on 6-well plates was performed as follows: (1) a total of 4.5 μg of plasmid DNA was diluted with serum-free DMEM up to 50 μl volume per well and incubated at room temperature for 5 min; (2) 10 μl of Lipofectamine 2000 was added to 40 μl serum-free DMEM for a total of 50 μl volume per well and incubated at room temperature for 5 min; (3) the diluted Lipofectamine 2000 solution was then added to the diluted DNA tube, flicked for thorough mixing, and incubated at room temperature for 20 min; (4) the DNA-Lipofectamine 2000 complex was then added to each well dropwise and evenly. Transfection of HEK293T cells seeded on 10-cm plates was performed as follows: (1) a total of 24 μg of plasmid DNA was diluted with serum-free DMEM up to 250 μl volume per well and incubated at room temperature for 5 min; (2) 60 μl of Lipofectamine 2000 was added to 190 μl of serum-free DMEM for a total of 250 μl volume per well and incubated at room temperature for 5 min; (3) the diluted Lipofectamine 2000 solution was then added to the diluted DNA tube, flicked for thorough mixing, and incubated at room temperature for 20 min; (4) the DNA-Lipofectamine 2000 complex was then added to each 10-cm plate dropwise and evenly.

MG132 (20 μM) or cycloheximide (15 μg/ml) was added 4 h prior to protein extraction in experiments examining Stat3 ubiquitination and protein stability in the presence of Hectd3.

### Protein extraction

Protein extraction of cells was performed as follows^68–70^. P8340 protease inhibitor cocktail (Cat#P8340, Sigma, Millipore-Sigma, MO, USA), cOmplete EDTA-free protease inhibitor cocktail (Cat# 11873580001, Roche Diagnostics, IN, USA), 1 mM NaF phosphatase inhibitor, 1 mM Na_3_VO_4_·2H_2_O phosphatase inhibitor, 1 mM β-glycerophosphate phosphatase inhibitor, and PR-619 deubiquitinase inhibitor (Cat# SI9619, LifeSensors, PA, USA) were added to cytoplasmic protein extraction buffer BN (15 mM Tris pH 7.5, 60 mM KCl, 5 mM MgCl_2_, 15 mM NaCl, 250 mM sucrose, 0.3% IGEPAL CA-630) and nuclear protein extraction buffer NEB (25 mM Tris pH 8, 250 mM NaCl, 10% glycerol, 0.2% IGEPAL CA-630). Primary mouse CD4^+^ T cells, CD8^+^ T cells, B220^+^ B cells, and CD11b^+^ cells or transfected HEK293T cells were vortexed in BN buffer, incubated on ice for 5 min, centrifuged at 3500 rpm at 4 °C, and then the supernatants were collected as cytoplasmic protein extract. The pellets were then vortexed in NEB buffer, incubated at 4 °C with rotation for 30 min, centrifuged at 13,000 rpm for 15 min at 4 °C, and then the supernatants were collected as nuclear protein extract. Cytoplasmic and nuclear protein extracts were combined as total protein for enrichment of ubiquitinated protein, immunoprecipitation, and immunoblot analysis.

For Malt1 substrate cleavage and CBM complex formation experiments, the cell lysis methods were as follows^40^. For cellular analyses, 2–3 × 10^6^ Jurkat T cells were lysed in co-immunoprecipitation (co-IP) buffer (25 mmM HEPES pH 7.5, 150 mM NaCl, 0.2% NP-40, 10% glycerol, 1 mM DTT, 10 mM sodium fluoride, 8 mM β-glycerophosphate, 300 µM sodium vanadate, and Roche protease inhibitor cocktail mix).

### Protein concentration measurement assay

The concentration of the protein samples was determined using DC Protein Assay (Cat#5000112, Bio-Rad Laboratories, CA, USA) according to the manufacturer’s protocol.

### Enrichment of ubiquitinated proteins

At least 700 μg of total protein from mouse primary CD4^+^ T cells was enriched for ubiquitinated protein with Ubiquitinated Protein Enrichment Kit (Cat#662200, Calbiochem, Millipore-Sigma, MO, USA) or TUBE2 Agarose (Cat#UM402, LifeSensors, PA, USA), following the manufacturer’s protocol.

### Immunoprecipitation

Immunoprecipitation were performed as follows^68–70^. At least 700 μg of total protein was used for immunoprecipitation. Protein A Sepharose (PAS) CL-4B (Cat#17096303, GE Healthcare Bio-Sciences, PA, USA) was washed ten times with TD150 buffer (10 mM HEPES, 150 mM NaCl, 10% glycerol, 1 mM DTT, 0.025% IGEPAL CA-630) and resuspended in equal volume of TD150 to obtain 50% PAS. A total of 100 μl of 50% PAS was needed for each sample. For pre-clearing of each sample, 50 μl of 50% PAS was added to each total protein sample, rotated at 4 °C for 1 h, centrifuged at 5000 rpm for 5 min at 4 °C, and then the supernatant was collected as pre-cleared samples. A total of 2 μg of Anti-Flag M2 antibody (Cat#F1804, Sigma, Millipore-Sigma, MO, USA) or Anti-Xpress antibody (Cat#R910-25, Invitrogen, Thermo Fisher Scientific, PA, USA) or Stat3 (124H6) (Cat#9139, Cell Signaling Technology, MA, USA) or Malt1 (Cat#2494, Cell Signaling Technology, MA, USA) was added to pre-cleared samples, and then incubated at 4 °C with rotation for 30 min. A total of 40 μl of 50% PAS was then added to antibody-pre-cleared sample mix and incubated at 4 °C with rotation from 4 h to overnight. Immunoprecipitated protein samples were centrifuged at 5000 rpm for 5 min at 4 °C, washed with TD150 three times, then denatured using 4× Laemmli Sample Buffer (Cat#161-0747, Bio-Rad Laboratories, CA, USA) with 10% 2-mercaptoethanol and boiled for 5 min. Denatured samples were subjected to immunoblot analysis.

Two-step immunoprecipitation and ubiquitination assays were performed as the following. For the first-round immunoprecipitation, protein extracts were prepared by using Lysis buffer (50 mM Tris-Cl pH 7.4, 150 mM NaCl, 1% Triton X-100, 1 mM EDTA) supplemented with cOmplete EDTA-free protease inhibitor cocktail (Cat# 11873580001, Roche Diagnostics, IN, USA). Lysates were incubated with the anti-Flag (M2)-agarose affinity gel (Cat#A2220, Sigma, Millipore-Sigma, MO, USA) for 2 h. The immunoprecipitates were washed three times with Lysis buffer. For the second-round immunoprecipitation, the immunoprecipitates were denatured by boiling for 5 min in the Lysis buffer containing 1% SDS. The elutes were diluted 1:10 with Lysis buffer. The diluted elutes were re-immunoprecipitated with the anti-Flag (M2)-agarose affinity gel. After extensive wash, the immunoprecipitates were subjected to immunoblot analysis.

For CBM complex analysis, the following precipitation was used as follows^40^. CBM complex formation was monitored by StrepTactin pulldown (ST-PD): 2 × 10^7^ cells were lysed in co-IP buffer, lysate controls taken, mixed with 4× SDS-loading buffer and boiled for 5 min at 95 °C. StrepII-tagged proteins were pulled down with StrepTactin Sepharose beads (15 µl 1:1 suspension) at 4 °C overnight. StrepTactin beads were washed with co-IP buffer, 22 µl 2× SDS-loading buffer added, and boiled for 8 min at 95 °C. Lysates and precipitated proteins were separated by SDS polyacrylamide gel electrophoresis and analyzed by western blot.

### Immunoblot

Denatured protein samples were subjected to gel electrophoresis by loading into 7.5% (Cat#4568024, Bio-Rad Laboratories, CA, USA) or gradient gel (Cat#4568124, Bio-Rad Laboratories, CA, USA). Gel electrophoresis was performed at 100 V for 10 min, and then at 200 V for at least 40 min. Proteins were then transferred to 0.2 μm PVDF membrane (Cat#1704156, Bio-Rad Laboratories, CA, USA) using the Trans-Blot Turbo Transfer System (Cat#1704150). PVDF membrane with transferred protein was washed with 1× TBST for 5 min at room temperature and shaking, blocked with 5% skim milk in 1× TBST for 30 min at room temperature and shaking, then washed with 1× TBST for 5 min at room temperature and shaking. The following antibodies for immunoblot were diluted in 4% BSA-TBST as indicated: 1:1000 Anti-Flag M2 antibody (Cat#F1804, Sigma, Millipore-Sigma, MO, USA); 1:2500 Anti-Xpress antibody (Cat#460528, Invitrogen, Thermo Fisher Scientific, PA, USA); 1:2000 Anti-HA (12CA5) antibody (Cat#11583816001, Roche Diagnostics, IN, USA); 1:3000 GAPDH (14C10) (Cat#2118L, Cell Signaling Technology, MA, USA); 1:2000 Stat3 (79D7) (Cat#4904S, Cell Signaling Technology, MA, USA); 1:2000 Phospho-Stat3 (Tyr705) (D3A7) (Cat#9145, Cell Signaling Technology, MA, USA); 1:1000 Malt1 (Cat#2494, Cell Signaling Technology, MA, USA); 1:1000 Hectd3 (Cat#A304-924A, Bethyl Laboratories, TX, USA); 1:1000 ubiquitin (P4D1) (Cat#3936, Cell Signaling Technology, MA, USA); 5 μg/ml RORγt (AFKJS-9) (Cat#14-6988-82, eBioscience, Thermo Fisher Scientific, PA, USA). Diluted antibodies were added as appropriate to the blocked blot and incubated at 4 °C with tilting rotation overnight. Immunoblots were washed with 1× TBST three times for 5 min at room temperature and shaking, and then blocked with 5% skim milk in 1× TBST for 10 min at room temperature and shaking. The following secondary antibodies for immunoblot were diluted 1:5000 in 5% skim milk-TBST: Peroxidase AffiniPure Goat Anti-Mouse IgG (H + L) (Cat#115-035-003, Jackson ImmunoResearch, PA, USA); Peroxidase AffiniPure Goat Anti-Rabbit IgG (H + L) (Cat#111-035-003, Jackson ImmunoResearch, PA, USA); Peroxidase AffiniPure Goat Anti-Rat IgG (H + L) (Cat#112-035-003, Jackson ImmunoResearch, PA, USA). Diluted secondary HRP-conjugated antibodies were added to appropriate immunoblots, and incubated at room temperature for 1 h with shaking. Immunoblot were washed once with 1× TBST, then twice with 1× TBS. Immunoblots were visualized with Lumi-Light Western Blotting Substrate (Cat#12015200001, Roche, IN, USA) and SuperSignal^TM^ West Femto Maximum Sensitivity Substrate (Cat#34095, Thermo Fisher Scientific, PA, USA) using a ChemiDoc Touch Gel Imaging System (Cat#1708370, Bio-Rad Laboratories, CA, USA). Immunoblot images were analyzed with Image Lab software (Bio-Rad Laboratories, CA, USA).

For Malt1 KO Jurkat reconstitution experiments, the following protocol was used. For αCD3/CD28 stimulation of Jurkat T cells, the following antibodies were used: mouse anti-human CD3 (1.0 µg/ml), mouse anti-human CD28 (3.3 µg/ml), rat anti-mouse IgG1 (1.7 µg/ml), and rat anti-mouse IgG2a (1.7 µg/ml) (all BD Pharmingen). For western blot and FACS studies, the following antibodies were used (dilution 1:1000 except when stated otherwise): anti-CARD11 (ID12) (Cell Signaling Technologies); anti-BCL10 (H-197), anti-HOIL-1 (H-1), anti-MALT1 (B12), anti-β-Actin (C4; 1:10,000), anti-CYLD (E-10) (all Santa Cruz Biotechnology); anti-hCD2-APC (RPA-2.10, eBioscience, FACS 1:200); horseradish peroxidase (HRP)-conjugated secondary antibodies (Jackson ImmunoResearch, 1:5000). For immunodetection, the proteins were transferred onto PVDF membranes using an electrophoretic semi-dry blotting system. Prior to primary antibody addition, membranes were blocked with 5% BSA or milk in PBS-T (0.1% Tween) for 1 h at RT. Membranes were incubated with primary antibody overnight at 4 °C as indicated above in 2.5% BSA or milk in PBS-T. For detection of primary antibodies, HRP-coupled secondary antibodies (in 1.25% BSA or milk in PBS-T) were applied to the membranes for 1 h at room temperature, and HRP visualized by enhanced chemiluminescence (ECL) with LumiGlo reagent (Cell Signaling Technologies) according to the manufacturer’s protocol.

### Anti-FLAG immunoprecipitation for mass spectrometry

Protein extracts from HEK293T cells co-transfected with HA-Ub and Flag-Stat3 vectors, or HA-Ub, Flag-Stat3 and Xpress-Hectd3 vectors, or HA-Ub and Flag-Malt1A vectors, or HA-Ub, Flag-Malt1A, and Xpress-Hectd3 vectors were subjected to immunoprecipitation using ANTI-FLAG M2 Affinity Gel (Cat#A2220, Sigma, Millipore-Sigma, MO, USA) according to the manufacturer’s protocol. Anti-FLAG Affinity Gel was washed with 1× TBS ten times and then added to protein samples, and the protein sample-Anti-FLAG Affinity Gel suspension was incubated at 4 °C with rotation for 4 h. The immunoprecipitated samples were centrifuged at 8200×*g* for 1 min at 4 °C, and then washed three times with 1× TBS. Immunoprecipitated samples were eluted with 0.1 M glycine HCl, pH 3.5, following the manufacturer’s protocol (Cat#A2220, Sigma, Millipore-Sigma, MO, USA).

### Sample preparation for tandem mass spectrometry

Samples were treated with 1 mM dithiothreitol (DTT) in 40 mM NH_4_HCO_3_ at 25 °C for 30 min, followed by 5 mM iodoacetamide (IAA) at 25 °C for 30 min in the dark. Protein was digested with 1:100 (w/w) lysyl endopeptidase (Wako) at 25 °C for 2 h and further digested overnight with 1:50 (w/w) trypsin (Promega) at 25 °C. Resulting peptides were desalted with a Sep-Pak C18 column (Waters) and dried under vacuum.

### Tandem mass spectrometry LC–MS/MS

Derived peptides were resuspended in 10 µl of loading buffer (0.1% formic acid, 0.03% trifluoroacetic acid, 1% acetonitrile). Peptide mixtures (2 µl) were separated on a self-packed C18 (1.9 µm, Dr. Maisch, Germany) fused silica column (25 cm × 75 µM internal diameter (ID); New Objective, Woburn, MA) by a Dionex Ultimate 3000 RSLCNano and monitored on a Fusion mass spectrometer (Thermo Fisher Scientific, San Jose, CA). Elution was performed over a 120-min gradient at a rate of 300 nl/min with buffer B ranging from 3 to 80% (buffer A: 0.1% formic acid in water, buffer B: 0.1 % formic in acetonitrile). The mass spectrometer cycle was programmed to collect at the top speed for 3-s cycles. The MS scans (400−1500 *m*/*z* range, 200,000 AGC, 50 ms maximum ion time) were collected at a resolution of 120,000 at *m*/*z* 200 in profile mode and the HCD MS/MS spectra (2 *m*/*z* isolation width, 30% collision energy, 10,000 AGC target, 35 ms maximum ion time) were detected in the ion trap. Dynamic exclusion was set to exclude previous sequenced precursor ions for 30 s within a 10-ppm window. Precursor ions with +1 and +8 or higher charge states were excluded from sequencing.

### Proteomics data analysis

The spectra were searched using Proteome Discoverer 2.0 against human Uniprot database (90,300 target sequences). Searching parameters included fully tryptic restriction and a parent ion mass tolerance (±20 ppm). Methionine oxidation (+15.99492 Da), asparagine and glutamine deamidation (+0.98402 Da), lysine ubiquitination (+114.04293 Da), and protein N-terminal acetylation (+42.03670) were variable modifications (up to three allowed per peptide); cysteine was assigned a fixed carbamidomethyl modification (+57.021465 Da). Percolator was used to filter the peptide spectrum matches to a false discovery rate of 1%. In-house software was used to compile the data across all project runs. The mass spectrometry proteomics data have been deposited to the ProteomeXchange Consortium with the dataset identifier PXD011756.

### Quantification and statistical analyses

Measurements were taken from distinct samples in the case of cells in culture and from individual mice in case of mice. Differences between groups were determined by a two-tailed Student’s *t* test assuming unequal variance and *p* < 0.05 was considered significant. For comparison of EAE clinical scores, Mann–Whitney two-tailed test was performed and *p* < 0.05 was considered significant.

### Reporting Summary

Further information on experimental design is available in the Nature Research Reporting Summary linked to this article.

## Supplementary information

Supplementary Information

Reporting Summary

Source Data

## Data Availability

The mass spectrometry proteomics data have been deposited to the ProteomeXchange Consortium with the dataset identifier PXD011756. All relevant reagents and data supporting the findings of this study are available from the corresponding author upon request.
