# Supplementary material for: Hectd3 promotes pathogenic Th17 lineage through Stat3 activation and Malt1 signaling in neuroinflammation
Source: Nat Commun. 2019 Feb 11;10:701. doi: 10.1038/s41467-019-08605-3 (PMC6370850; doi:10.1038/s41467-019-08605-3)
Supplement: Supplementary file 1 — Supplementary Information [file 41467_2019_8605_MOESM1_ESM.pdf]

Hectd3 promotes pathogenic Th17 lineage through Stat3 activation and Malt1 signaling in neuroinflammation

Cho et al.

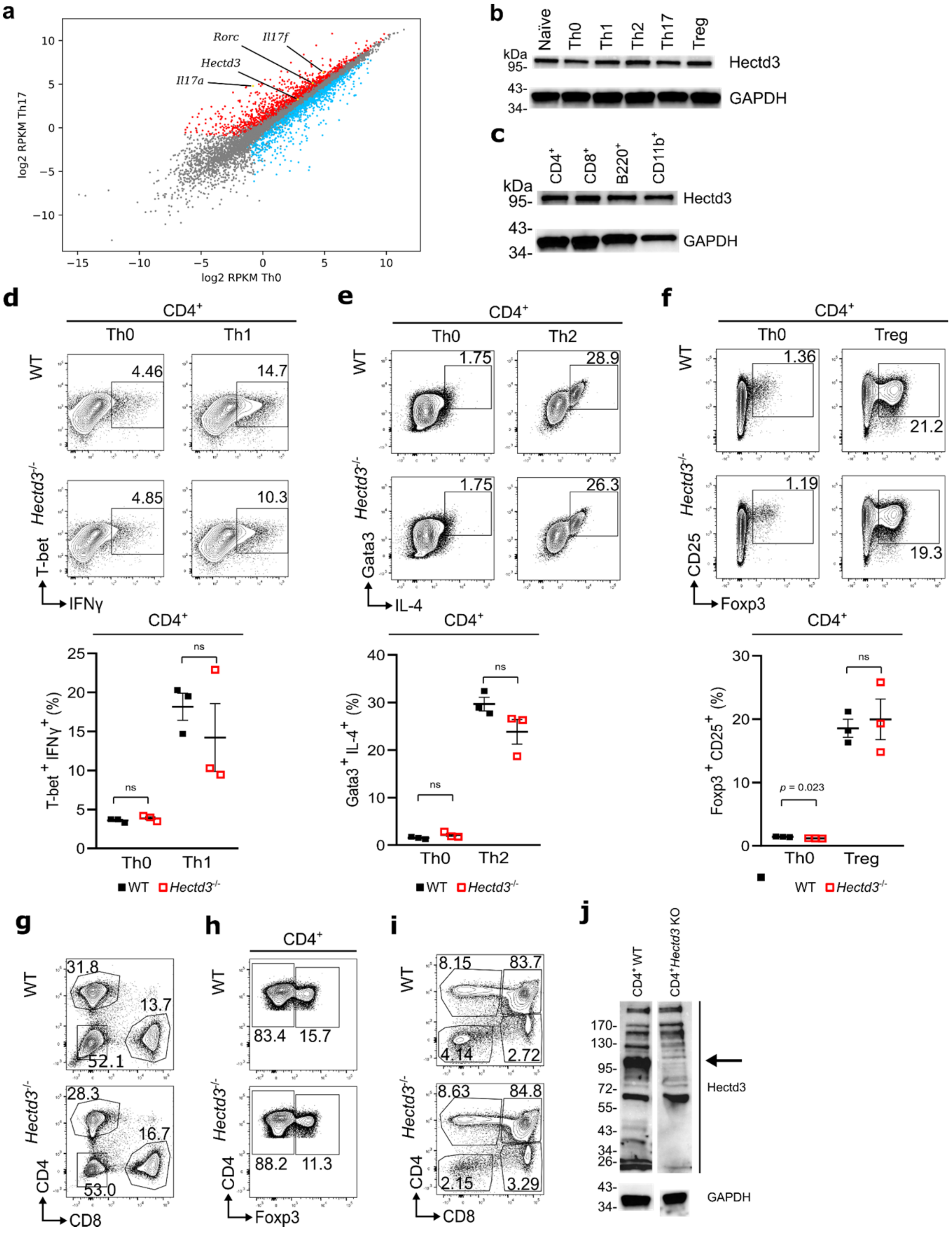
a
Rorc
Il17f
Hectd3
Il17a
log2 RPKM Th17
log2 RPKM Th0
b
Naïve
Th0
Th1
Th2
Th17
Treg
kDa
95-
43-
34-
Hectd3
GAPDH
c
CD4+
CD8+
B220+
CD11b+
d
CD4+
Th0
Th1
WT
Hectd3-/-
4.46
14.7
4.85
10.3
T-bet
IFNγ
T-bet+ IFNγ+ (%)
ns
WT
e
Th2
1.75
28.9
1.75
26.3
Gata3
IL-4
Gata3+ IL-4+ (%)
f
Treg
1.36
21.2
1.19
19.3
CD25
Foxp3
Foxp3+ CD25+ (%)
p = 0.023
g
31.8
13.7
52.1
28.3
16.7
53.0
CD4
CD8
h
83.4
15.7
88.2
11.3
i
8.15
83.7
4.14
2.72
8.63
84.8
2.15
3.29
j
CD4+ WT
CD4+ Hectd3 KO
170-
130-
95-
72-
55-
26-

Supplementary Figure 1

Thymic and peripheral T cell populations and polarization into Th1, Th2, and Treg conditions are normal in the absence of Hectd3. a RNA-sequencing of *in vitro* polarized mouse wild type Th17 and Th0 cells as reported by Ciofani, *et al*[1]**.** Data are shown as a scatter plot of log2 of reads per kilobase per million mapped reads (RPKM). Blue dots represent >2 z-score downregulated genes, and red dots represent >2 z-score upregulated genes in Th17 cells relative to Th0 cells. Genes of interest are indicated and marked in yellow (upregulated) with the following z-scores: *Hectd3* 2.42, *Rorc* 8.93, *Il-17a* 6.71, *Il-17f* 7.05 (from [1]). **b** Representative immunoblot of Hectd3 and GAPDH in WT naïve and *in vitro* polarized $CD4^+$ T cells. **c** Representative immunoblot of Hectd3 and GAPDH in WT purified $CD4^+$ T, $CD8^+$ T, $B220^+$, and $CD11b^+$ cells. (**b-c)** Data are representative of two independent experiments. **d-f** Representative flow cytometry analysis and frequencies of T-bet and IFNγ **(d),** Gata3 and IL-4 **(e)** and Foxp3 and CD25 **(f)** from *in vitro* polarized *Hectd3*$^{-/-}$ and WT $CD4^+$ T cells under Th0, Th1 **(d)**, Th2 **(e)**, and Treg **(f)** conditions. Purified $CD4^+$ T cells were cultured and polarized as described in Materials and Methods. **(d-f)** Data are presented as mean ± SEM and derived from two independent experiments; *p* values were obtained from Student's t test. Gating strategy shown in Supplemental Figure 9. **g-i** Representative flow cytometry analysis of $CD4^+$ and $CD8^+$ T cell populations **(g)** and Treg cells **(h)** in peripheral lymph nodes, and of thymic populations **(i)** in *Hectd3*$^{-/-}$ and WT mice at steady state. **j** Representative immunoblot of Hectd3 and GAPDH in purified $CD4^+$ T of *Hectd3*$^{-/-}$ and WT $CD4^+$ mice. Representative of two independent experiments. Arrow indicates specific Hectd3 band. Source data are provided as a Source Data file.

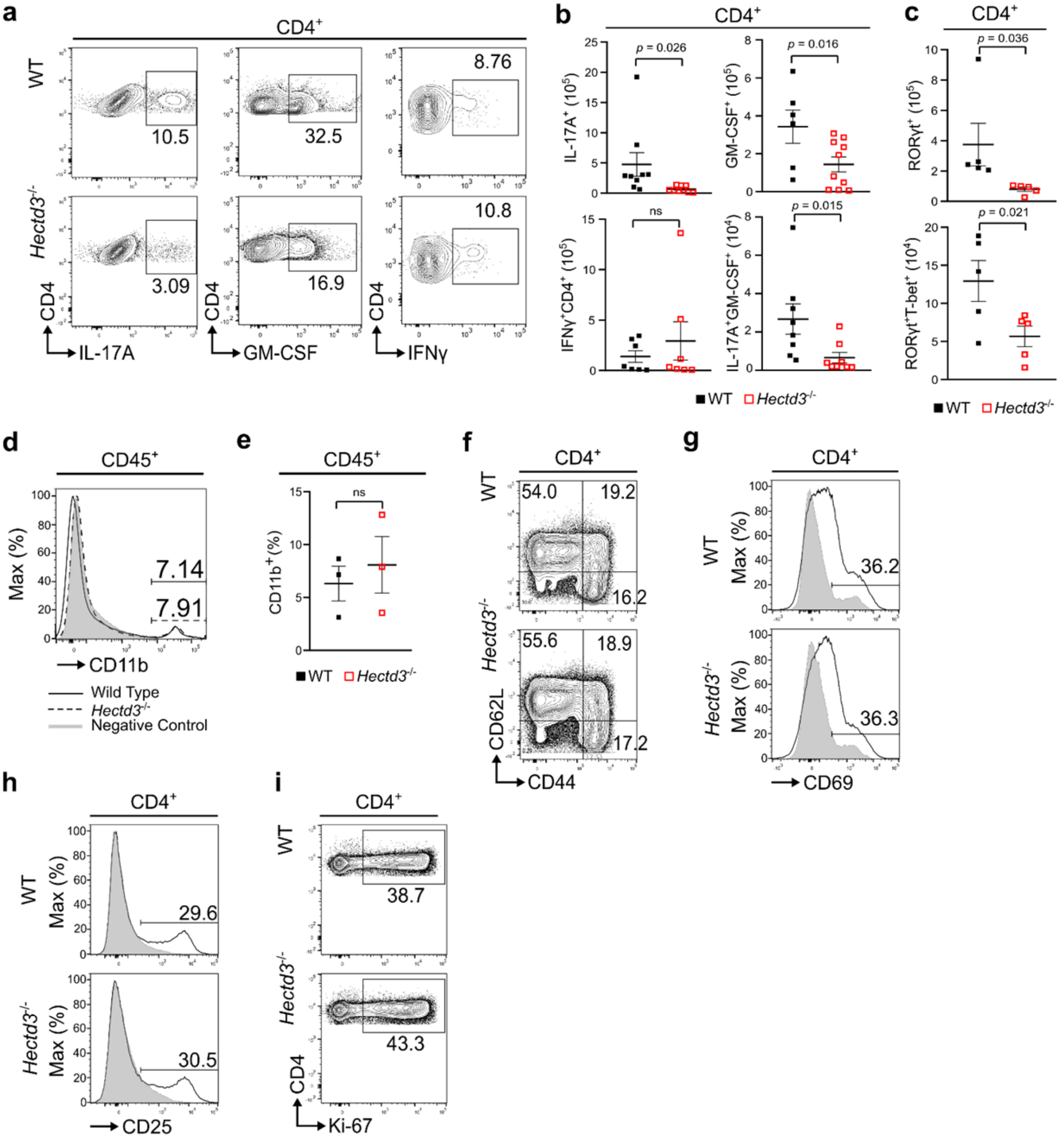
a
CD4+
WT
Hectd3-/-
10.5
32.5
8.76
3.09
16.9
10.8
CD4
IL-17A
GM-CSF
IFNγ
b
CD4+
p = 0.026
p = 0.016
ns
p = 0.015
IL-17A+ (10^5)
GM-CSF+ (10^5)
IFNγ+CD4+ (10^5)
IL-17A+GM-CSF+ (10^4)
WT
Hectd3-/-
c
CD4+
p = 0.036
p = 0.021
RORγt+ (10^5)
RORγt+T-bet+ (10^4)
WT
Hectd3-/-
d
CD45+
Max (%)
7.14
7.91
CD11b
Wild Type
Hectd3-/-
Negative Control
e
CD45+
ns
CD11b+ (%)
WT
Hectd3-/-
f
CD4+
WT
Hectd3-/-
54.0
19.2
16.2
55.6
18.9
17.2
CD62L
CD44
g
CD4+
WT
Hectd3-/-
Max (%)
36.2
36.3
CD69
h
CD4+
WT
Hectd3-/-
Max (%)
29.6
30.5
CD25
i
CD4+
WT
Hectd3-/-
38.7
43.3
CD4
Ki-67

Supplementary Figure 2

IL-17A, GM-CSF, and RORγt are diminished in *Hectd3$^{-/-}$* CD4^{+} T cells of mice induced with EAE but activation and proliferation are not altered. a Representative flow cytometry analysis of intracellular IL-17A, GM-CSF, and IFNγ in CD4^{+} T cell from the draining lymph nodes dLNs of *Hectd3$^{-/-}$* and WT mice, 13 days following EAE induction; n=5 per group from three independent experiments. Gating strategy shown in Supplemental Figure 9. **b** Absolute numbers of IL-17A^{+}, GM-CSF^{+}, IFNγ^{+}, and IL-17A^{+}GM-CSF^{+} CD4^{+} T cells in the draining lymph nodes of *Hectd3$^{-/-}$* and wild type mice 13 days following EAE induction. **c** Absolute numbers of RORγt^{+} CD4^{+} T cells and RORγt^{+} T-bet^{+} CD4^{+} T cells in dLNs of *Hectd3$^{-/-}$* and WT mice, 13 days following EAE induction. **d-e** Flow cytometry analysis of CD11b from the CNS of *Hectd3$^{-/-}$* and WT mice 22 days following EAE induction. Gating strategy was first on CD45^{+} T cells. **f** Flow cytometry analysis of CD44^{+}, CD62L^{+}, and CD44^{+}CD62L^{+} CD4^{+} T cells from dLNs of *Hectd3$^{-/-}$* and WT mice 7 days following EAE induction. Gating strategy show in Supplemental Figure 9. **g-h** Flow cytometry analysis of surface CD69 **(g)** and CD25 **(h)** on CD4^{+} T cells from dLNs of *Hectd3$^{-/-}$* and WT mice 7 days following EAE induction. Gating strategy shown on Supplemental Figure 9. **i** Flow cytometry analysis of intranuclear Ki-67 in CD4^{+} T cells from dLNs of *Hectd3$^{-/-}$* and WT mice 7 days following EAE induction. Gating strategy shown in Supplemental Figure 9. **(a,d,f-i)** Data (n = 6) are representative of three independent experiments. **(b,c,e)** Data are presented as mean ± SEM; *p* values was obtained from Student's t test. Source data are provided as a Source Data file.

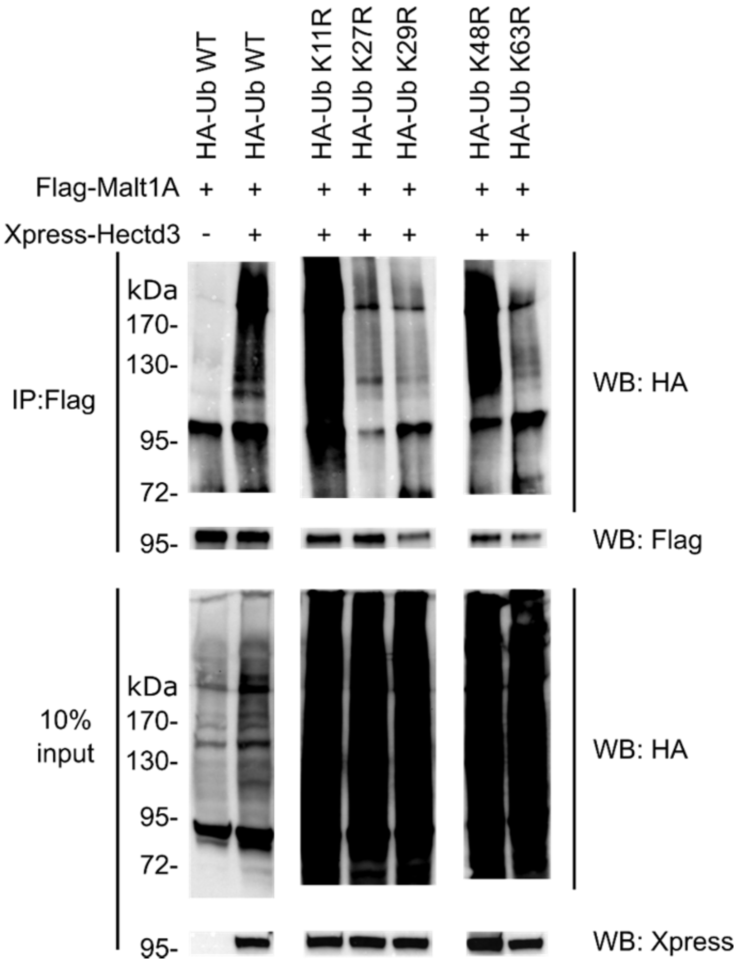

Supplementary Figure 3

Hectd3 promotes K27- and K29-linked polyubiquitin on Malt1A. Immunoblot of HA and Flag following two-step Flag immunoprecipitation of protein extracts from HEK293T cells co-transfected with the indicated HA-Ub K to R mutants, Flag-Malt1A and Xpress-Hectd3. HA-Ub K to R single mutant denote that the indicated lysine residue is mutated to arginine in the HA-Ub. Immunoblots are representative of two independent experiments. Source data are provided as a Source Data file.

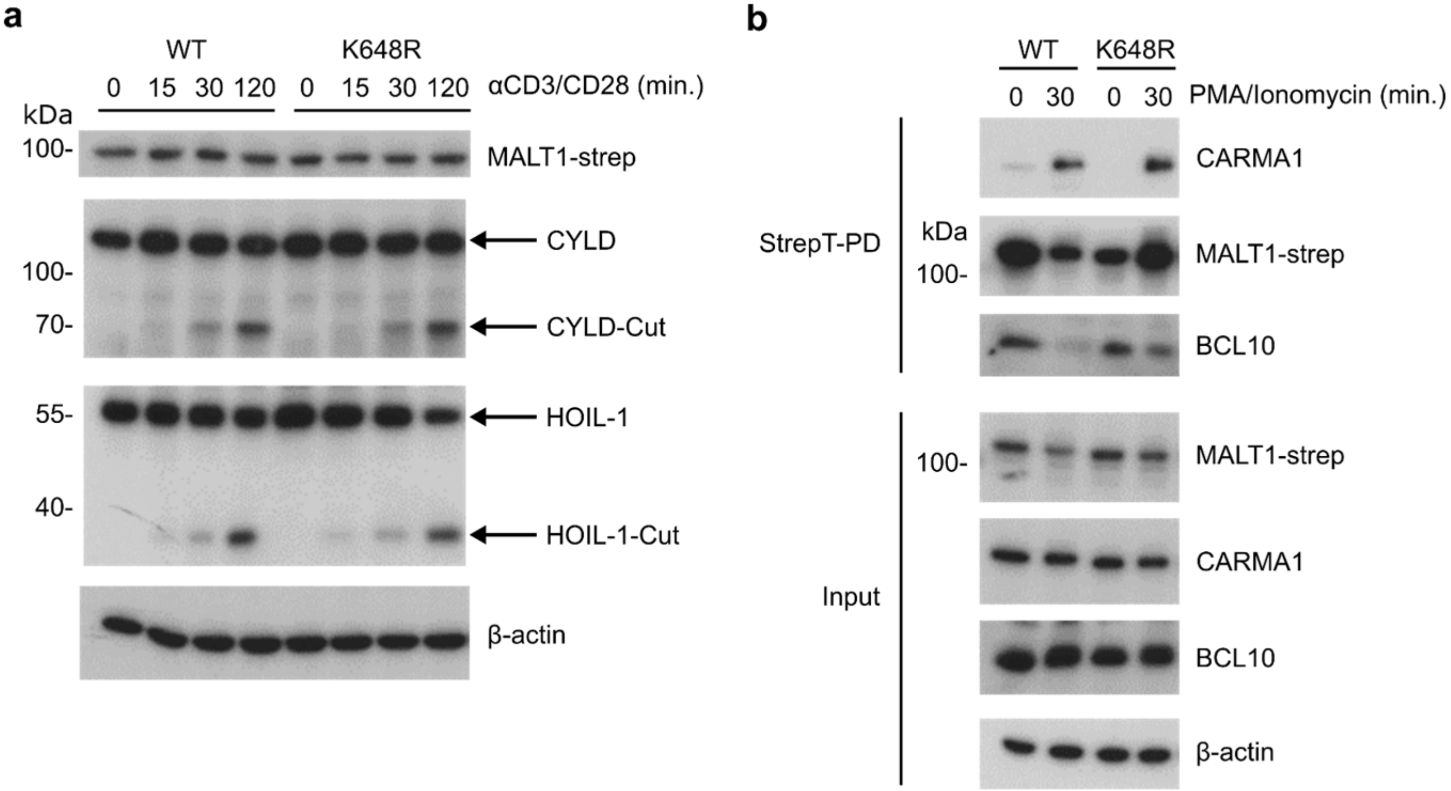

Supplementary Figure 4

Malt1A K648R does not affect Malt1 substrate cleavage and CMB complex formation in Jurkat cells. a Immunoblot of MALT1-strep, CYLD, HOIL-1, and β-actin of protein extracts from MALT1KO Jurkat cells transduced with MSCV-Malt1A WT or MSCV-Malt1A K648R following the indicated duration of αCD3/CD28 stimulation. **b** Immunoblot of CARMA1, MALT1-strep, BCL10, and β-actin following StrepTactin pulldown (StepT-PD) of protein extracts from MALT1KO Jurkat cells transduced with MSCV-Malt1A WT or MSCV-Malt1A K648R. **(a-b)** Immunoblots are representative of two independent experiments. Source data are provided as a Source Data file.

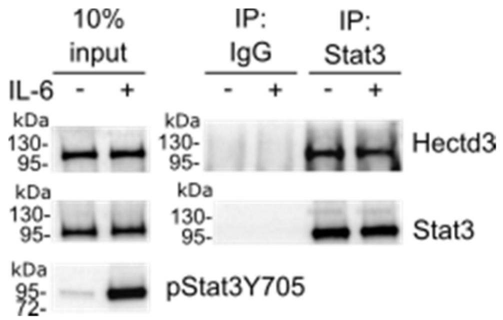

Supplementary Figure 5

Hectd3 associates with Stat3 CD4$^+$ T cells regardless of IL-6 stimulation. Immunoblot of Hectd3, Stat3 and pStat3 Y705 following Stat3 immunoprecipitation of protein extracts from WT CD4$^+$ T cells stimulated or not with IL-6 (50 ng/ml) for 30 min. Representative of 3 independent experiments. Source data are provided as a Source Data file.

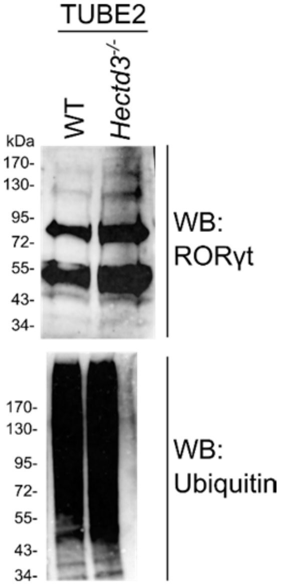

Supplementary Figure 6

Hectd3 does not promote polyubiquitination of RORγt in EAE CD4$^+$ T cells. Immunoblot for RORγt and ubiquitin following polyubiquitinated protein enrichment with TUBE2 of extracts from the dLN CD4$^+$ T cells of *Hectd3*$^{-/-}$ and WT mice 13 days following EAE induction. Representative of two independent experiments. Source data are provided as a Source Data file.

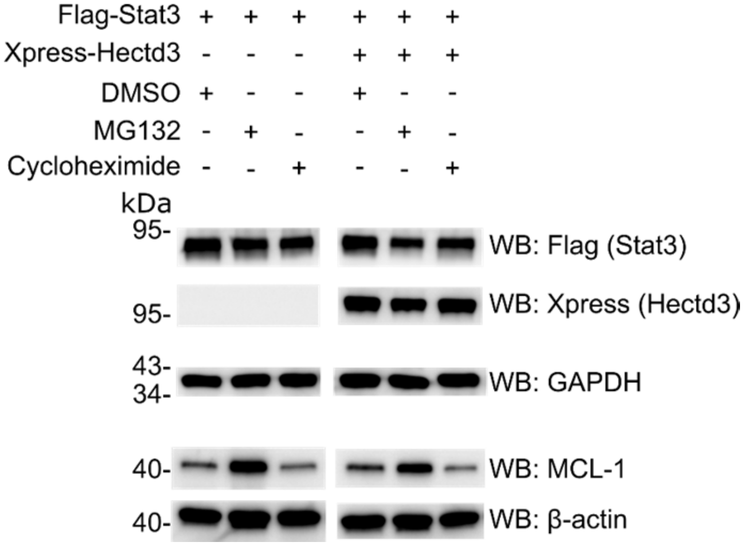

Supplementary Figure 7

Hectd3 does not target Stat3 for proteasomal degradation. Immunoblot of Flag, Xpress, GAPDH, MCL-1, and β-actin of protein extracts from HEK293T cells co-transfected as indicated with Flag-Stat3 and Xpress-Hectd3 and treated for 4 hours with 20 μM MG132 or 15 μg/mL cycloheximide prior to protein extraction. Immunoblots are representative of two independent experiments. Source data are provided as a Source Data file.

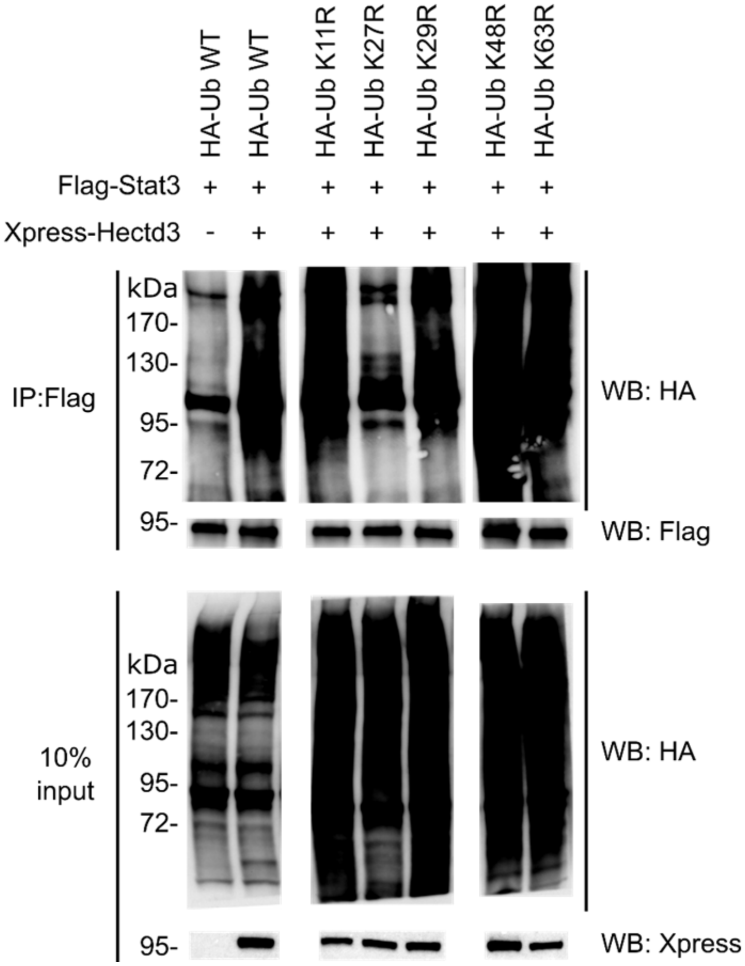

Supplementary Figure 8

Hectd3 promotes K27-linked polyubiquitin chain on Stat3. Immunoblot of HA and Flag following two-step Flag immunoprecipitation of protein extracts from HEK293T cells co-transfected as indicated with HA-Ub K to R mutants, Flag-Stat3, and Xpress-Hectd3. HA-Ub K to R single mutants denote that the indicated lysine residue is mutated to arginine in the HA-Ub. Immunoblot is representative of two independent experiments. Source data are provided as a Source Data file.

a

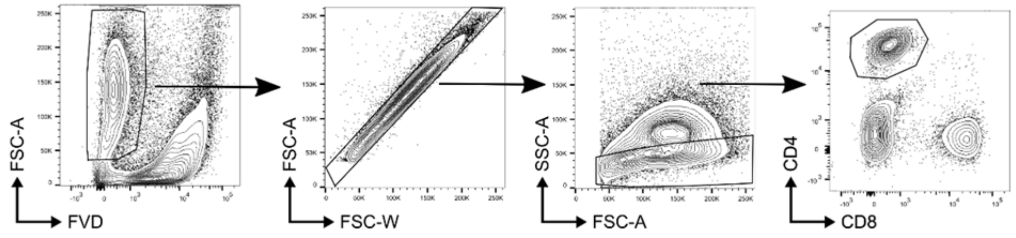

Supplementary Figure 9. Gating strategy. $CD4^+$ T cells from the dLNs and CNS were gated on live, single cell lymphocytes, CD4 and CD8. Cytokines, surface markers and transcription factors were shown the $CD4^+$ T cells. A similar strategy of gating was used for *in vitro* polarization on CD4+ T cells, as well as for transduced $CD4^+$ T cells with retroviruses. Transduced $CD4^+$ T cells were additionally gated on GFP, or CD90.1.

					Stat3							Stat3+Hectd3						
Protein Name	Accession	Calc PI	#AA	MW [kDA]	Coverage [%]	#Peptides	#Unique Peptides	Area	HT Sequest Score	Confidence (Sequest HT)	#PSMs	Coverage [%]	#Peptides	#Unique Peptides	Area	HT Sequest Score	Confidence (Sequest HT)	#PSMs
E3 ubiquitin-protein ligase HECTD3	Q5T447	5.64	861	97.05	0	0	0	0	0	n/a	0	14.9825784	10	10	2.80E+07	28.93	High	13
Signal transducer and activator of transcription 3	P40763	6.3	770	88	82.14286	4	1	1E+09	97.49	High	31	71.2987013	53	50	1.449E+10	1440.17	High	602

Supplementary Table 1

Mass spectrometry-based analysis of Stat3 and Hectd3. HEK293T cells were co-transfected with HA-Ub and Flag-Stat3 (Stat3), or co-transfected with HA-Ub, Flag-Stat3 and Xpress-Hectd3 (Stat3+Hectd3), and immunoprecipitated with anti-Flag antibodies, followed by trypsin digestion and Tandem Mass Spectrometry, as described in Material and Methods. Confidence was assigned by Sequest HT algorithm, which was used in conjunction with q-value calculator and posterior error probability (PEP) test to assign peptide confidence. Sequence coverage, number of identified peptides, number of unique peptides, sum of area of all peptides identified, sum of Sequest HT score, confidence and number of PSMs is shown per each protein in each sample. False discovery rate was 0.011% for peptide groups in Flag IP sample from HEK293T cells co-transfected Flag-Stat3 and Xpress-Hectd3 vectors (Stat3+Hectd3) and 0.01% for Flag IP sample from HEK293T cells transfected with Flag-Stat3 vector (Stat3).

Confidence Sequest HT	Sequence	Modifications	Qvality PEP	Qvality q-value	# PSMs	Master Protein Accessions	# Missed Cleavages	Theo. MH+ [Da]	Area	XCorr Sequest HT	Percolator q-Value Sequest HT	Percolator PEP Sequest HT
High	DFPAVDSVLVK		0.0099	0.0023	2	Q5T447	0	1189.64632	7630788	2.05	0.008364	0.04593
High	EQVAAMQAGLLK		0.0013	0.0003	1	Q5T447	0	1258.68238	11545471	2.41	0.000253	0.001138
High	FIAEGIIDQGGGFR		0.0121	0.0031	1	Q5T447	0	1479.75905	24512584	2.17	0.002746	0.009738
High	LEGTDPEVLYR		0.0004	0	1	Q5T447	0	1291.65286	14586199	2.23	0	0.000369
High	LVPIDTPNHLQR	1xDeamidated [N8]	0.0003	0	2	Q5T447	0	1403.76414	45346712	2.71	0	0.000261
High	RVVVYGGEGDNLKK	1xDeamidated [N11]	0.0236	0.005	1	Q5T447	2	1534.82238	unable to calculate	2.66	0.004143	0.0187
High	RVVVYGGEGDNLKK		5E-05	0	1	Q5T447	2	1533.83837	5882221	2.91	0	4.68E-05
High	TANQGNGTGEAR	1xDeamidated [N6]	0.0008	0	1	Q5T447	0	1176.52397	3043183	2.55	0	0.000653
High	VLLPVWEAEGLGLR		0.0479	0.0094	1	Q5T447	0	1551.88934	15091793	1.19	0.007354	0.03743
High	VVPQAVLDLLTWQELEK		0.0108	0.0025	1	Q5T447	0	1981.10045	1225384	2.43	0.002379	0.008753
High	WEQVVDLTYSHR		0.0006	0	1	Q5T447	0	1532.74922	3455042	2.78	0	0.00052

Supplementary Table 2

Mass spectrometric analysis of Hectd3 peptides. HEK293T cells were co-transfected with HA-Ub and Flag-Stat3 (Stat3), or co-transfected with HA-Ub, Flag-Stat3 and Xpress-Hectd3 (Stat3+Hectd3), and immunoprecipitated with anti-Flag antibodies, followed by trypsin digestion and Tandem Mass Spectrometry, as described in Material and Methods. XCorr values were calculated by utilizing Sequest HT algorithm and used for calculating peptide confidence. Percolator was additionally used to discriminate between correct and incorrect peptide matches. Statistical analysis by q-value and posterior error probability (PEP) were used to increase confidence in the peptide identifications at a false discovery rate of 0.011% for peptide groups.

					Stat3					Stat3+Hectd3				
**Peptide**	**Modifications**	**Position (residue number)**	**Modification Position**	**Protein Accession Number(s)**	**XCorr Sequest HT**	**Percolator PEP**	**Percolator q-value**	**Confidence (Sequest HT)**	**#PSMs**	**XCorr Sequest HT**	**Percolator PEP**	**Percolator q-value**	**Confidence (Sequest HT)**	**#PSMs**
TLkSQGDMQDLNGNNQSVTR	K3(GlyGly)	178-197	**K180**	P40763						2.58	0.0242	0.00511	High	1
TLkSQGDMQDLnGNNQSVTR	K3(GlyGly); N12(Deamidated)	178-197	**K180**;N189	P40763						2.24	0.0124	0.00275	High	1
TLkSQGDmQDLnGnNQSVTR	K3(GlyGly); M8(Oxidation); N12(Deamidated); N14(Deamidated)	178-197	**K180;**M185;N189;N191	P40763						2.91	0.00364	0.00136	High	1
TLkSQGDMQDLNGnNQSVTR	K3(GlyGly); N14(Deamidated)	178-197	**K180**;N191	P40763						2.25	0.021	0.00492	High	1
DISGkTQIQSVEPYTK	K5(GlyGly)	627-642	**K631**	P40763	2.96	0.0497	0.00618	High	1					
AILSTkPPGTFLLR	K6(GlyGly)	596-609	**K601**	P40763	2.36	0.00015	0	High	1					

Supplementary Table 3

Mass spectrometry-based analysis of ubiquitinated Stat3 peptides. HEK293T cells were co-transfected with HA-Ub and Flag-Stat3 (Stat3), or co-transfected with HA-Ub, Flag-Stat3 and Xpress-Hectd3 (Stat3+Hectd3), and immunoprecipitated with anti-Flag antibodies, followed by trypsin digestion and Tandem Mass Spectrometry, as described in Material and Methods. XCorr values were calculated by utilizing Sequest HT algorithm and used for calculating peptide confidence. Percolator was additionally used to discriminate between correct and incorrect peptide matches. Statistical analysis by q-value and posterior error probability (PEP) were used to increase confidence in the peptide identifications at a false discovery rate of 0.011 % for Flag IP sample from HEK293T cells co-transfected Flag-Stat3 and Xpress-Hectd3 vectors (Stat3+Hectd3) and 0.01% for Flag IP sample from HEK293T cells transfected with Flag-Stat3 vector (Stat3).

Name of primer	Sequence
Hectd3 forward	5’-GGAAGATCTCCTCCTCCTGCCGGCCCTGGC-3’
Hectd3 reverse	5’- CCGCTCGAGTCACTCCTCCCAGGGACT-3’
Stat3 forward	5’-AGTCAGACTGAGATCTCCACCATGGCTCAGTGGAACCAGCTG-3’
Stat3 reverse	5’- AGTCTCAGTGCTCGAGTTATCACATGGGGGAGGTAGCACACTC-3’
Stat3 K180R forward	5’- CAAAACCCTCAGGAGCCAAGGAG-3’
Stat3 K180R reverse	5’- TAGTTGAAATCAAAGTCGTCCTG-3’
Malt1A K648R forward	5’- AGATGCAAATAGAGGCACACCTG-3’
Malt1A K648R reverse	5’- TTTGGATCAATATCTAGATCAAGTG-3’
Stat3 Δaa2-130 forward	5’-CACCCAACAGCCGCCGTAG-3’
Stat3 Δaa2-130 reverse	5’-CATGGCGATCGCGGCGGC-3’
Stat3 Δaa131-320 forward	5’-TTCGTGGTGGAGCGGCAG-3’
Stat3 Δaa131-320 reverse	5’-GTTGGCCTGGCCCCCTTG-3’
Stat3 Δaa320-480 forward	5’-AACATGCTGACCAATAAC-3’
Stat3 Δaa320-480 reverse	5’-ACTCTTCATTAAGTTTCTGAAC-3’
Stat3 Δaa481-574 forward	5’-TATATCTTGGCCCTTTGG-3’
Stat3 Δaa481-574 reverse	5’-ATACCACAGGATTGATGC-3’
Stat3 Δaa576-679 forward	5’-GAGGAGGCATTTGGAAAG-3’
Stat3 Δaa576-679 reverse	5’-ATACTTTTTCACAAGGTCG-3’
Stat3 Δaa671-770 forward	5’-ACGCGTACGCGGCCGCTC-3’
Stat3 Δaa671-770 reverse	5’-CTCCTTGGGAATGTCGGGGTAGAGG-3’
Hectd3 Δaa2-109 forward	5’-GAGCTGTGCAACGGCCAC-3’
Hectd3 Δaa2-109 reverse	5’-AGGAGGAGGCAGATCCTC-3’
Hectd3 Δaa110-397 forward	5’-GGCACTGACCCAGAAGTACTATAC-3’
Hectd3 Δaa110-397 reverse	5’-CTCGCCAGTGGTGCGCAC-3’
Hectd3 Δaa398-511 forward	5’-TATGAGAAGCCCTTGGAC-3’
Hectd3 Δaa398-511 reverse	5’-TTCCAGGCGTGGATATCG-3’
Hectd3 Δaa512-861 forward	5’-TGACTCGAGTCTAGAGGG-3’
Hectd3 Δaa512-861 reverse	5’-CTTGTCAGAAGGCTTGAG-3’
HA-Ub K6R forward	5’-GATCTTCGTGaggACCCTGACCGGCAAGACCATC-3’
HA-Ub K6R reverse	5’-TGCATGCCGCCGCCGCCG-3’
HA-Ub K6,11R forward	5’-CCTGACCGGCagaACCATCACCC-3’
HA-Ub K6,11R reverse	5’-GTCCTCACGAAGATCTGC-3’
HA-Ub K6,11,27R forward	5’-CGAGAACGTGaggGCCAAGATCC-3’
HA-Ub K6,11,27R reverse	5’-ATGGTGTCGCTGGGCTCC-3’

HA-Ub K6,11,27,29R forward	5'-CGTGAGGGCCagaATCCAGGACA-3'
HA-Ub K6,11,27,29R reverse	5'-TTCTCGATGGTGTCGCTG-3'
HA-Ub K6,11,27,29,33R forward	5'-AATCCAGGACagaGAGGGCATCCCC-3'
HA-Ub K6,11,27,29,33R reverse	5'-CTGGCCCTCACGTTCTCG-3'
HA-Ub K6,11,27,29,33,48R forward	5'-CTTCGCCGGCagaCAGCTGGAGG-3'
HA-Ub K6,11,27,29,33,48R reverse	5'-ATCAGTCTCTGCTGGTCG-3'
HA-Ub K6,11,27,29,33,48,63R forward	5'-CAACATCCAGagaGAGAGCACCCTG-3'
HA-Ub K6,11,27,29,33,48,63R reverse	5'-TAGTCGCTCAGGGTTCTG-3'
HA-Ub K6 only forward	5'-GATCTTCGTGaagACCCTGACCGGCAGAACC-3'
HA-Ub K6 only reverse	5'-TGCATGCCGCCGCCGCCG-3'
HA-Ub K11 only forward	5'-CCTGACCGGCaagACCATCACCC-3'
HA-Ub K11 only reverse	5'-GTCCTCACGAAGATCTGC-3'
HA-Ub K27 only forward	5'-CGAGAACGTGaagGCCAGAATCC-3'
HA-Ub K27 only reverse	5'-ATGGTGTCGCTGGGCTCC-3'
HA-Ub K29 only forward	5'-CGTGAGGGCCaagATCCAGGACA-3'
HA-Ub K29 only reverse	5'-TTCTCGATGGTGTCGCTG-3'
HA-Ub K33 only forward	5'-AATCCAGGACaagGAGGGCATCCCC-3'
HA-Ub K33 only reverse	5'-CTGGCCCTCACGTTCTCG-3'
HA-Ub K48 only forward	5'-CTTCGCCGGCaagCAGCTGGAGG-3'
HA-Ub K48 only reverse	5'-ATCAGTCTCTGCTGGTCG-3'

Supplementary Table 4

Primers used for PCR cloning and mutagenesis

References

1. Ciofani, M. *et al.* A validated regulatory network for Th17 cell specification. *Cell* **151**, 289-303 (2012).
