# Supplementary material for: Hectd3 promotes pathogenic Th17 lineage through Stat3 activation and Malt1 signaling in neuroinflammation
Source: Nat Commun. 2019 Feb 11;10:701. doi: 10.1038/s41467-019-08605-3 (PMC6370850; doi:10.1038/s41467-019-08605-3)
Supplement: Supplementary file 2 — Reporting Summary [file 41467_2019_8605_MOESM2_ESM.pdf]

nature research

Corresponding author(s): Dorina Avram

Last updated by author(s): 01.14.19

Reporting Summary

Nature Research wishes to improve the reproducibility of the work that we publish. This form provides structure for consistency and transparency in reporting. For further information on Nature Research policies, see Authors & Referees and the Editorial Policy Checklist.

Statistics

For all statistical analyses, confirm that the following items are present in the figure legend, table legend, main text, or Methods section.

n/a	Confirmed	
☐	☒	The exact sample size (*n*) for each experimental group/condition, given as a discrete number and unit of measurement
☐	☒	A statement on whether measurements were taken from distinct samples or whether the same sample was measured repeatedly
☐	☒	The statistical test(s) used AND whether they are one- or two-sided<br>*Only common tests should be described solely by name; describe more complex techniques in the Methods section.*
☐	☒	A description of all covariates tested
☐	☒	A description of any assumptions or corrections, such as tests of normality and adjustment for multiple comparisons
☐	☒	A full description of the statistical parameters including central tendency (e.g. means) or other basic estimates (e.g. regression coefficient) AND variation (e.g. standard deviation) or associated estimates of uncertainty (e.g. confidence intervals)
☐	☒	For null hypothesis testing, the test statistic (e.g. *F*, *t*, *r*) with confidence intervals, effect sizes, degrees of freedom and *P* value noted<br>*Give P values as exact values whenever suitable.*
☒	☐	For Bayesian analysis, information on the choice of priors and Markov chain Monte Carlo settings
☒	☐	For hierarchical and complex designs, identification of the appropriate level for tests and full reporting of outcomes
☒	☐	Estimates of effect sizes (e.g. Cohen's *d*, Pearson's *r*), indicating how they were calculated

Our web collection on statistics for biologists contains articles on many of the points above.

Software and code

Policy information about availability of computer code

Data collection	BD FACS Diva
Data analysis	FlowJo, Microsoft Office Excel, GraphPad Prism, Proteome Discoverer 2.0, Image Lab 6.0.1, Percolator, IDEAS 6.1

For manuscripts utilizing custom algorithms or software that are central to the research but not yet described in published literature, software must be made available to editors/reviewers. We strongly encourage code deposition in a community repository (e.g. GitHub). See the Nature Research guidelines for submitting code & software for further information.

Data

Policy information about availability of data

All manuscripts must include a data availability statement. This statement should provide the following information, where applicable:

- Accession codes, unique identifiers, or web links for publicly available datasets
- A list of figures that have associated raw data
- A description of any restrictions on data availability

The mass spectrometry proteomics data have been deposited to the ProteomeXchange Consortium with the dataset identifier PXD011756 (http://proteomecentral.proteomexchange.org/cgi/GetDataset?ID=PXD011756). All relevant reagents and data supporting the findings of this study are available from the corresponding author upon request.

Field-specific reporting

Please select the one below that is the best fit for your research. If you are not sure, read the appropriate sections before making your selection.

☒ Life sciences ☐ Behavioural & social sciences ☐ Ecological, evolutionary & environmental sciences

For a reference copy of the document with all sections, see nature.com/documents/nr-reporting-summary-flat.pdf

Life sciences study design

All studies must disclose on these points even when the disclosure is negative.

Sample size	Sample sizes were not pre-calculated. Sample sizes were determined by using three pairs of mice at least in order to determine statistical significance. Transfection experiments were repeated three times independently, as recommended by Vaux, D., et al. EMBO Rep. 2012 Apr; 13(4): 291–296.
Data exclusions	No data were excluded from the analyses.
Replication	Experiments were repeated three times independently to ensure reproducibility. Attempts at replication were predominantly successful.
Randomization	This is not relevant, all mice are genetically identical except for the deletion of Hectd3 in the knock-out (KO) mice. Cells from cell lines are identical upon plating.
Blinding	Blinding was done for EAE scoring, as Jonathan Cho induced the disease in mice and Theodore Drashansky scored without knowing the groups.

Reporting for specific materials, systems and methods

We require information from authors about some types of materials, experimental systems and methods used in many studies. Here, indicate whether each material, system or method listed is relevant to your study. If you are not sure if a list item applies to your research, read the appropriate section before selecting a response.

Materials & experimental systems

n/a	Involved in the study	
☐	☒	Antibodies
☐	☒	Eukaryotic cell lines
☒	☐	Palaeontology
☐	☒	Animals and other organisms
☒	☐	Human research participants
☒	☐	Clinical data

Methods

n/a	Involved in the study	
☒	☐	ChIP-seq
☐	☒	Flow cytometry
☒	☐	MRI-based neuroimaging

Antibodies

Antibodies used

anti-mouse CD4,Biolegend,100447,GK1.5,B245638
anti-mouse/human RORγt,Invitrogen,17-6988-82,AFKJS-9,1936933
anti-mouse/human T-bet,Invitrogen,25-5825-82,4B10,4277988
anti-mouse/human IL-17A,Invitrogen,48-7177-82,eBio17B7,4331772
anti-mouse GM-CSF,Biolegend,505406,MP1-22E9,4290464
anti-mouse IFNγ,Biolegend,505806,XMG1.2,B243512
anti-mouse CD25,Biolegend,102016,PC61,4289562
anti-mouse/human Foxp3,Invitrogen,48-5773-82,FJK-16s,4303738
anti-mouse CD8α,Biolegend,100708,53-6.7,multiple
anti-mouse IL-23R,BDBiosciences,744372,O78-1208,5321848
anti-mouse CD196 (CCR6),Biolegend,129819,29-2L17,B198149
anti-mouse CD29,Biolegend,102222,HMβ1-1,B205106
anti-mouse/human MALT1,Cell Signaling Technology,2494S,#2494,2
Goat anti-rabbit IgG,Invitrogen,A-11008,N/A,2018309
anti-mouse/human pStat3 (Tyr705),Cell Signaling Technology,4324S,#4324,15
anti-mouse/human CD44,Invitrogen,17-0441-83,IM7,4283622
anti-mouse CD62L,Biolegend,104408,MEL-14,B242686
anti-mouse CD69,Invitrogen,45-0691-82,H1.2F3,E08350-1634
anti-mouse/human Ki-67,Invitrogen,11-5698-82,SolA15,4283595
anti-mouse/human NF-κB p65,Cell Signaling Technology,4764S,C22B4,8
anti-mouse/human RelB,Cell Signaling Technology,#4922,C1E4,3
anti-GFP,Invitrogen,A-21311,N/A,1891008

anti-mouse/human CD90.1 (Thy1.1),Invitrogen,25-0900-82,HIS51,E07586-1631
anti-mouse CD3,BioX Cell,BE0002,17A2,5597/0415
anti-mouse CD28,BioX Cell,BE0015-1,37.51,5375/1114
anti-mouse IL-4,BioX Cell,BE0045,11B11,5872/1215
anti-mouse IFNγ,BioX Cell,BE0055,XMG1.2,3344/0212
anti-mouse IL-12,BioX Cell,BE0052,R1-5D9,909
anti-Ub Agarose TUBE 2,Life Sensors,UM402,N/A,TR-42832.001
anti-flag M2 monoclonal,Sigma,F1804-1MG,N/A,SLBT7654
anti-Xpress,Invitrogen,R910-25,N/A,2008680
anti-mouse/human Stat3 ,Cell Signaling Technology,9139S,124H6,10
anti-HA,Sigma,11666606001,12CA5,multiple
anti-mouse/human GAPDH,Cell Signaling Technology,2118S,14C10,10
anti-mouse/human Stat3,Cell Signaling Technology,4904S,79D7,7
anti-mouse/human pStat3 (Tyr705),Cell Signaling Technology,9145S,D3A7,31
anti-mouse/human HECTD3,Bethyl Laboratories,A304-924A-T,N/A,multiple
anti-ubiquitin,Cell Signaling Technology,3936S,P4D1,16
anti-mouse/human RORγt,Invitrogen,14-6988-82,AFKJS-9,E11084-1631
Peroxidase AffiniPure Goat Anti-Mouse IgG (H+L),Jackson ImmunoResearch Laboratories,115-035-003,N/A,multiple
Peroxidase AffiniPure Goat Anti-Rabbit IgG (H+L),Jackson ImmunoResearch Laboratories,111-035-003,N/A,15-11
Peroxidase AffiniPure Goat Anti-Rat IgG (H+L),Jackson ImmunoResearch Laboratories,112-035-003,N/A,multiple
anti-human CD3,BDBiosciences,550368,UCHT1,multiple
anti-human CD28,BDBiosciences,556620,CD28.2,multiple
anti-mouse IgG1,BDBiosciences,553445,A85-3,multiple
anti-mouse IgG2a,BDBiosciences,553446,R11-89,multiple
anti-mouse/human CARD11,Cell Signaling Technology,4435S,1D12,multiple
anti-mouse/human Bcl10,Santa Cruz Biotechnology,sc-5611,H-197,A101
anti-mouse/human RBCK1,Santa Cruz Biotechnology,sc-393754,H-1,multiple
anti-human MALT1,Santa Cruz Biotechnology,sc-46677,B-12,C2807
anti-mouse/human β-Actin,Santa Cruz Biotechnology,sc-130656,N-21,C2515
anti-mouse/human cylindromatosis 1,Santa Cruz Biotechnology,sc-74435,E-10,multiple
anti-mouse/human CD2,Invitrogen,17-0029-42,RPA-2.10,multiple

Validation

All antibodies were validated by the manufacturers, as per their protocols. Biolegend, Invitrogen, Cell Signaling Technology, Bethyl Laboratories, Santa Cruz Biotechnology all provide staining examples on the antibody page.

Eukaryotic cell lines

Policy information about cell lines

Cell line source(s)	HEK293T cells (Cat#CRL-3216, ATCC, VA, USA), Platinum-E (Plat-E) retroviral packaging cells (Cat#RV-101, Cell Biolabs, CA, USA), EL4 cells (Cat# TIB-39, ATCC, VA, USA), and Jurkat, (Cat# TIB-152, ATCC, VA, USA) were used.
Authentication	ATCC has authenticated each of the cell lines they sold to us: HEK293T, EL4, and Jurkat. Cell Biolabs has authenticated the Platinum-E retroviral packaging cells as a potent retrovirus packaging cell line.
Mycoplasma contamination	The cell lines were not tested for mycoplasma contamination.
Commonly misidentified lines (See ICLAC register)	Not applicable

Animals and other organisms

Policy information about studies involving animals; ARRIVE guidelines recommended for reporting animal research

Laboratory animals	Species used: mouse, strain: C57BL/6NTac, sex: male and female, age: 10-20 weeks old
Wild animals	Not applicable
Field-collected samples	Not applicable
Ethics oversight	All protocols were approved by the University of Florida Institutional Animal Care and Use Committee.

Note that full information on the approval of the study protocol must also be provided in the manuscript.

Flow Cytometry

Plots

Confirm that:

☐ The axis labels state the marker and fluorochrome used (e.g. CD4-FITC).

☒ The axis scales are clearly visible. Include numbers along axes only for bottom left plot of group (a 'group' is an analysis of identical markers).

☒ All plots are contour plots with outliers or pseudocolor plots.

☒ A numerical value for number of cells or percentage (with statistics) is provided.

Methodology

Sample preparation	Leukocytes were isolated from brain and spinal cord. Mice were euthanized with CO2, hepatic portal vein was severed, and left ventricle of heart was perfused with at least 10 ml ice-cold PBS. Perfused brain and spinal cord were dissected out and placed in gentleMACS C tubes (Cat#130-093-237, Miltenyi Biotec Inc., CA, USA) containing 3 ml of RPMI-1640 supplemented with 1% L-glutamine, 1% non-essential amino acids, 1% sodium pyruvate, 1% penicillin/streptomycin, 0.01M HEPES, 220 µM 2-mercaptoethanol, and 1% FBS (1% RPIMI). Brain and spinal cord tissue were then dissociated using gentleMACS Dissociator (Cat#130-093-235, Miltenyi Biotec Inc., CA, USA) setting m_brain_03.01, m_brain_02_02, and m_brain_01_02 for C tube. Dissociated brain and spinal cord were pressed through 70 µm nylon mesh (Cat#22363548, Fisher Scientific, ThermoFisher Scientific, PA, USA) with 1% RPMI up to a volume of 7 ml total. 100% isotonic Percoll was prepared by mixing 46.25 ml Percoll (Cat#17-0891-01, GE Healthcare Bio-Sciences, PA, USA) with 3.6 ml 10X HBSS (Cat#20-023-CV, Corning, NY, USA), and 0.6 ml 7.5% sodium bicarbonate. 100% isotonic Percoll was diluted to 70% isotonic Percoll with 1% RPMI. 3 ml of 100% isotonic Percoll was added to the 7 ml brain and spinal cord homogenate for a final 30% Percoll brain and spinal cord homogenate. The 10 ml 30% Percoll brain and spinal cord homogenate was then underlaid with 2 ml 70% isotonic Percoll using a glass Pasteur pipette. The 30-70% layered Percoll was centrifuged for 30 minutes, 500 g at room temperature without brake. The 30/70% interphase was collected with a transfer pipette following centrifugation, and washed with at least 3 fold volume of 1% RPMI then centrifuged for 10 minutes at 350 g, 4°C for collecting of leukocytes. Spleen and lymph nodes were pressed through 40 µm nylon mesh (Cat#22363547, Fisher Scientific, ThermoFisher Scientific, PA, USA).
Instrument	Flow cytometry was performed on a BD LSR II, model number: 338639
Software	Flow cytometry data were acquired using BD FACS DIVA software. All data were analyzed using FlowJo (TreeStar).
Cell population abundance	Purity of the samples was determined on the BD LSR II using an aliquot of the sorted sample, sorted samples were all greater 95% purity.
Gating strategy	For the experiments without transduction: live cells were gated using Fixable Viability Dye (negative) and FSC, single cells were gated on the diagonal of FSC-W and FSC-A, lymphocytes were gated on SSC-A (less than 50k) against FSC-A, CD4 and CD8 T cells were gated appropriately afterwards. For transduction experiments: live cells were gated using Fixable Viability Dye (negative) and FSC, single cells were gated on the diagonal of FSC-W and FSC-A, CD4 T cells were gated on CD4 (above 10^3) and FSC-A.

☒ Tick this box to confirm that a figure exemplifying the gating strategy is provided in the Supplementary Information.
